# Extracellular vesicles as tools and targets in therapy for diseases

**DOI:** 10.1038/s41392-024-01735-1

**Published:** 2024-02-05

**Authors:** Mudasir A. Kumar, Sadaf K. Baba, Hana Q. Sadida, Sara Al. Marzooqi, Jayakumar Jerobin, Faisal H. Altemani, Naseh Algehainy, Mohammad A. Alanazi, Abdul-Badi Abou-Samra, Rakesh Kumar, Ammira S. Al-Shabeeb Akil, Muzafar A. Macha, Rashid Mir, Ajaz A. Bhat

**Affiliations:** 1 Watson-Crick Centre for Molecular Medicine, Islamic University of Science and Technology, Awantipora, Kashmir 192122 India; 2 Department of Human Genetics-Precision Medicine in Diabetes, Obesity and Cancer Program, Sidra Medicine, Doha, Qatar; 3 Qatar Metabolic Institute, Academic Health System, Hamad Medical Corporation, Doha, Qatar; 4 Department of Medical Laboratory Technology, Prince Fahad Bin Sultan Chair for Biomedical Research, Faculty of Applied Medical Sciences, University of Tabuk, Tabuk, Saudi Arabia; 5 School of Biotechnology, Shri Mata Vaishno Devi University, Katra, India

**Keywords:** Tumour biomarkers, Drug development

## Abstract

Extracellular vesicles (EVs) are nano-sized, membranous structures secreted into the extracellular space. They exhibit diverse sizes, contents, and surface markers and are ubiquitously released from cells under normal and pathological conditions. Human serum is a rich source of these EVs, though their isolation from serum proteins and non-EV lipid particles poses challenges. These vesicles transport various cellular components such as proteins, mRNAs, miRNAs, DNA, and lipids across distances, influencing numerous physiological and pathological events, including those within the tumor microenvironment (TME). Their pivotal roles in cellular communication make EVs promising candidates for therapeutic agents, drug delivery systems, and disease biomarkers. Especially in cancer diagnostics, EV detection can pave the way for early identification and offers potential as diagnostic biomarkers. Moreover, various EV subtypes are emerging as targeted drug delivery tools, highlighting their potential clinical significance. The need for non-invasive biomarkers to monitor biological processes for diagnostic and therapeutic purposes remains unfulfilled. Tapping into the unique composition of EVs could unlock advanced diagnostic and therapeutic avenues in the future. In this review, we discuss in detail the roles of EVs across various conditions, including cancers (encompassing head and neck, lung, gastric, breast, and hepatocellular carcinoma), neurodegenerative disorders, diabetes, viral infections, autoimmune and renal diseases, emphasizing the potential advancements in molecular diagnostics and drug delivery.

## Introduction

**Fig. 1:**
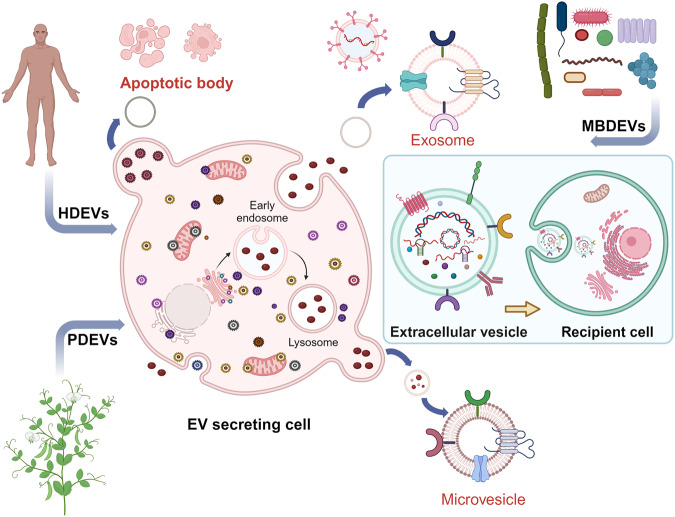
Visual depiction of the variety and sources of EVs. EVs encapsulate an array of bioactive entities, including proteins, nucleic acids, and lipids, which not only form structural components but also bear specific cellular signatures. Cells from diverse tissue origins employ EVs as vehicles for intercellular communication, releasing them into adjacent body fluids. In humans, a notable proportion of EVs emanate from stem cells. Beyond humans, various organisms, from plants to bacteria, also actively produce and release EVs into their environment

**Fig. 2:**
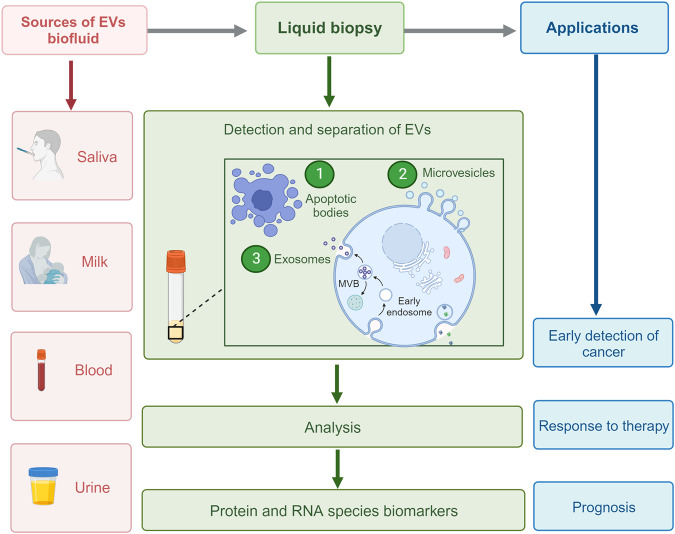
EVs are enriched in body fluids. This figure highlights the ubiquity of EVs across various body fluids. Liquid biopsy, which enables the non-invasive capture and analysis of EVs from fluids, including saliva, milk, blood, and urine, stands at the forefront of advancements in cancer diagnosis and prognosis prediction. The clinical relevance of EVs extends to monitoring therapeutic responses and forecasting disease outcomes. Their widespread presence in biofluids positions EVs as invaluable tools for refining patient management in oncology

Extracellular vesicles (EVs) represent a heterogeneous collection of lipid bilayer-enclosed particles, actively synthesized and secreted by a myriad of cell types into the extracellular milieu. Their secretion is a pervasive mechanism observed across all domains of life, encompassing both prokaryotes and eukaryotes, and it occurs under a range of conditions, from physiological to pathological states. While historically dismissed as mere cellular debris with limited relevance, current research has illuminated their pivotal role as bioactive carriers. These vesicles serve as conduits for transporting diverse cellular constituents, facilitating intricate cellular communication and mediating a plethora of biological processes.^1^ EVs carry a wide range of cargo, including proteins such as cell surface receptors, signaling proteins, transcription factors, enzymes, and extracellular matrix proteins.^2^ They also contain lipids and nucleic acids (such as miRNA, mRNA, and DNA) that can be transferred from parent to recipient cells, mediating intercellular communication and molecular transfer.^3^ EVs have been found to contribute to pathological diseases such as heart disease, neurodegenerative diseases, and cancer.^4^ EVs encompass various subtypes classified by their synthesis and release mechanisms, including exosomes, apoptotic blebs, and other EV subgroups.^5^ They can also be classified based on the originating cell type, for example, platelet-derived, endothelial cell-derived, or the physiological state of the cells, e.g., “oncosomes” discharged from cancer cells; “prostasomes” originated from the prostate. Microvesicles, exosomes, and apoptotic bodies are the main entities of EVs (Fig. 1),^6,7^ but recent research has identified additional types, such as large oncosomes, migrasomes,^8^ ectosomes,^9^ exomeres, supermeres, and membrane particles (Table 1). EVs are extensively distributed and have been detected in all human bodily fluids, including mother milk, cerebrospinal fluid, urine, saliva, and blood, both in healthy and pathological conditions (Fig. 2). Notably, the nature of the fluid, associated diseases, and the prevailing disease conditions correlate intricately with the EVs’ quantity, tissue provenance, molecular composition, and inherent functional traits.

**Table 1:** Features of extracellular vesicles

EV subtypes	Origin	Size (nm)	Biomarkers	Density (g/ml)	Mechanism	Refs.
Exosomes	Multivesicle body	50–150	CD9, CD63, Tsg101, CD81, ALIX, HSP70	1.13–1.19	Endosomes mature into late endosomes, forming multivesicular bodies (MVBs) with intraluminal vesicles that fuse with the plasma membrane for release (dependent or independent of ESCRT)	^88,595,596^
Microvesicles	Plasma membrane	100–1000	Integrins, Selectins, CD40, tissue factor	1.032–1.068	Calcium influx and cortical cytoskeleton remodeling cause direct plasma membrane budding and cleavage.	^595,597^
Apoptotic Bodies	Plasma membrane	100–5000	Annexin V, C3b, thrombospondin, Annexin A1, histone coagulation factor,	1.16–1.28	Cytoplasmic fragmentation during programmed cell death	^595,598,599^
Exomeres	Secreting from cells	≤50	TGFBI, ENO1 and GPC1	1.1–1.19	Cleavage of large cytoplasmic extensions from cell body	^600^
Migrasomes	Retraction Fibers	500–3000	Tspan4, CD63, Annexin A1	Unknown	Because of cell migration and actin polarization/ Migrasomes are formed at the tip or by bifurcation of the retraction fibers during migration	^601^
Oncosomes	the shedding of non-apoptotic plasma membrane blebbing	1000–10000	Cav-1 or ADP ribosylation factor 6	1.10–1.15	Released by cancer cells with amoeboid movement	^602,603^
Supermeres	Unknown	∼35 (<50)	TGFBI, ACE2, PCSK9,miR-1246, MET, GPC1 and AGO2, exRNA; miR-1246	Unknown	Unknown	^604,605^

Profiling proteins and Extracellular RNA (exRNA) in biofluids, notably urine and blood, holds substantial diagnostic and prognostic value. This could provide insights into the manifestations of either systemic or localized diseases. Given the remarkable capacity of EVs to encapsulate and preserve the molecular signature of their parent cells, they have emerged as potential treasure troves for biomarker discovery.^10^ A particular focus has been on human milk (HM), which is teeming with a spectrum of bioactive constituents pivotal for infant health. EVs in HM have been conjectured to play a role in protecting infants against conditions like necrotizing enterocolitis. Furthermore, EVs sourced from HM present a promising therapeutic avenue for neonates in scenarios where maternal breastfeeding is unfeasible and securing donor-expressed breast milk poses challenges. Nevertheless, a comprehensive, precise assessment of existing data on HM-sourced EVs remains an unmet need.^11^ In a parallel vein, the composition of salivary EVs has emerged as a potential biomarker reservoir, particularly for oral and systemic malignancies. Their presence and profile could serve as indicators for both localized and systemic diseases. These EVs can induce biological effects over long distances, as they can circulate throughout the body, not just locally in the microenvironment of the releasing cells.^12^ EVs play a pivotal role in preserving the homeostasis of various systems and organs within the body and in the onset and progression of a myriad of diseases, notably cancer and neurological disorders. Regardless of whether they arise from healthy tissues or diseased sites, EVs can act as carriers for pathogenic proteins and miRNAs, potentially facilitating the spread and intensification of certain diseases. One of the primary mechanisms through which EVs exert their influence is interacting with recipient cells. They accomplish this through binding to specific receptor molecules found on the cell surface. Among these receptors are tetraspanin proteins, integrins (ITGs), immunoglobulins, and proteoglycans. Such interactions not only foster communication between cells but also play a role in guiding the EVs to their targeted cells. Of particular note is the observation that cancerous cells tend to produce EVs in larger volumes and with a richer cargo content compared to their healthy counterparts, as evidenced in the literature.^13^ EVs bind to recipient cells through surface molecules, altering the target cells’ physiological state^14^ and influencing various aspects of cancer growth. They mediate critical pathways associated with cancer progression, known as “cancer hallmarks,”^15^ and play crucial roles in early and late processes related to tumor development and metastasis.^16^

In cancer, EVs form a supportive tumor microenvironment (TME) and pre-metastatic niches (PMN).^17^ Studies showed that highly aggressive forms of brain tumor cells had large amounts of EVs.^18^ The cargo of cancer-derived EVs is associated with advanced disease characteristics,^19^ such as metastasis, therapy resistance, and immune evasion.^20^ There is a need for the detection of EVs to diagnose and prevent common diseases early. Various methods detect and analyze EV subtypes and their cargo content, allowing differentiation between cancer-derived EVs and those from normal cells. EVs are frequently reported to be raised in the blood in response to chronic and acute inflammation associated with different diseases. Identifying and tracking EVs in the bloodstream can potentially revolutionize their use as biomarkers. Furthermore, EVs released by pathogen-infected cells and other common diseased cells can alter cellular biology, leading to cancer, immune suppression, and tissue damage during sepsis. Activated polymorphonuclear leukocytes from septic patients have been shown to produce EVs with increased adhesion molecules that can trigger the vascular endothelium, leading to endothelial injury and resultant organ dysfunction.^21^

The therapeutic and diagnostic potential of EVs is presently on the cusp of a significant breakthrough. Since their relatively recent discovery as crucial mediators in both physiological processes and disease progression, there has been an accelerated interest in harnessing these EVs for medical applications. Their inherent roles in cell-to-cell communication and molecular transfer underscore their relevance in biomedicine. The potential for clinical uses of EVs is currently at a turning point. As a result of their relatively recent identification as essential participants in physiology and disease, the utilization of these tiny vesicles for diagnostic and therapeutic reasons is fast-growing. EVs derived from cancer and affected cells offer new opportunities and biomarkers for diagnosing and predicting the prognosis of common diseases. These EVs are known to protect their cargo, making them valuable carriers for targeted drug delivery.^22^ Moreover, their role as drug carriers can be harnessed for chemotherapy and to evaluate the effectiveness of therapeutic drugs. Cancer researchers have invested considerable time and resources in understanding how intercellular communication mediated by EVs impacts different aspects of cancer growth, intending to develop novel approaches to combat the disease. Additionally, the therapeutic use of EVs secreted by various cell types, mainly stem and progenitor cells, offers significant advantages over using the parent cells.

This review delves deeply into the multifaceted world of EVs, offering a detailed exploration of their biogenesis, composition, and pivotal roles in physiology. A central theme of this review is the intricate relationship between EVs and their cargo, particularly miRNAs and proteins, and how these associations can influence the onset and progression of various cancers. We shed light on the latest advancements and strategies aimed at harnessing EVs for therapeutic interventions.

Furthermore, this review offers insights into the specific roles of EVs in head and neck squamous cell carcinoma (HNSCC), gastric cancer (GC), lung cancer (LC), and breast cancer (BC), illuminating how their presence and behavior can be leveraged for enhanced cancer management.

But the influence of EVs isn’t restricted to oncology alone. The review broadens its scope to delve into the significance of EVs in the realm of neurodegenerative diseases, diabetes, viral infections, autoimmune disorders, and renal diseases. By offering this comprehensive overview, we underscore the increasing importance of EVs in molecular diagnostics, therapeutics, and drug delivery systems, underscoring their potential to reshape our approach to disease detection, management, and treatment.

### Biogenesis and composition of extracellular vesicles

The terminology surrounding the biogenesis and heterogeneity of EVs has led to misconceptions and contradictions. In recent years, various mechanisms of EV biogenesis have been identified, including the involvement of the ESCRT complex, tetraspanins, sphingomyelinases, relocalization of phospholipids, and depolymerization of the actin cytoskeleton.^23^ In the intricate process of EV biogenesis, both exosomes and ectosomes undergo unique formation pathways. Recent studies have illuminated nuanced mechanisms involved in their biogenesis, highlighting the role of cellular components not previously appreciated. EVs formed through inward-budding vesicles within the endocytic system, known as exosomes, or through outward-budding vesicles at the plasma membrane are called Microvesicles (MVs).^24^ Exosomes are generated by the fusion of multivesicular bodies (MVBs) with the plasma membrane, regulated by molecules such as neutral sphingomyelinase 2 (nSMase2), endosomal sorting complex required for transport (ESCRT) complexes, syntenin, ALIX, tetraspanins, Rab proteins, and phospholipase D2.^25^ The MVs’ inward budding captures cytosolic material, including proteins and nucleic acids. New research suggests the involvement of the ESCRT machinery and ESCRT-independent pathways, including those mediated by lipid-dependent mechanisms.^26^ Additionally, the tetraspanin-enriched microdomains (TEMs) are now understood to play a crucial role in exosome biogenesis and cargo selection.^27^

MVs originate through the outward protrusion of the plasma membrane, a phenomenon observed during apoptosis, leading to the release of apoptotic bodies.^28^ This MV formation is orchestrated by intricate molecular pathways, commonly initiated by factors such as cellular stress or activation signals. Recent research has highlighted the significance of calcium influx, cytoskeleton reorganization, and the enzymatic functions of proteins, notably floppases, and scramblases, in ectosome biogenesis.^29^ Further deepening our understanding of EV biogenesis, the Ras-related protein Rab27a stands out as a pivotal player in the exosome secretion pathway.^30^ In another revealing discovery, the syndecan-syntenin-ALIX complex has been pinpointed as a key regulator of the biogenesis and dispatch of exosome-like EVs, broadening our comprehension of the molecular foundations governing EV creation.^31^ While there are fundamental processes shared across EV biogenesis in different cell types, it’s crucial to acknowledge that individual cellular environments and external stimuli can significantly influence the constitution and functionality of the EVs produced. Such intricacies imply that EV biogenesis is not merely a standardized process but a nuanced, tightly controlled, and context-sensitive means of cellular dialog. Notably, even with variances in their biogenesis processes and originating membranes, exosomes and MVs exhibit analogous functionalities once they enter the extracellular space.^29^

Several cellular components have been identified as participating in EV biogenesis. Proteins such as CD63, CD81, CD9, ALIX, TSG101, syntenin, ubiquitin, clathrin, VPS32, VPS4, ERK, PLD, and ARF6 are important in EV biogenesis.^26^ EV biogenesis also requires energy and cofactors such as ATP and NADPH. ATP plays a crucial role in providing energy for the active processes involved in EV biogenesis, including V-type proton ATPase subunit B (Atp6v1b2), RNA helicase DDX25 (Ddx25), and Sodium/potassium transporting ATPase subunits alpha-1 and 3 (Atp1a1, Atp1a3).^32^ ATP is also involved in cytoskeleton rearrangement processes required for EV biogenesis.^33^ Tumors express the ATP receptor P2X7 receptor (P2X7R) that contributes to EV biogenesis and secretion in tumor pathogenesis.^34,35^ GAPDH, a glycolytic enzyme, is associated with EV biogenesis, secretion, and assembly. Overexpression of human GAPDH in certain cells, specifically in secondary adult cells (SCs), promotes intraluminal vesicle (ILVs) formation,^36^ and Rab11-exosome biogenesis in glutamine depletion cancer cells.^37^ Another protein, Arrdc4, is involved in EV biogenesis in the epithelial cells of the epididymis and is necessary for sperm motility and fertility.^38^ This study was further supported by adding fractions enriched in EVs from wild-type epididymal cells to Arrdc4^–/-^ sperm, leading to in vitro restoration of a two-cell embryo.^38^ Actin cytoskeleton regulating proteins, such as cortactin, promote exosome secretion and influence tumor growth, cancer cell invasion, and motility.^39^ Cancer cells employ various proteins, including ARF1,^40^ ARF6,^41^ and RhoA,^42^ to bud EVs off from the plasma membrane. Increased levels of activated ARF6 have been observed in melanoma and BC cells, resulting in higher EV secretion.^41^ Proteins in coordinating molecular machinery are also crucial for EV biogenesis and secretion. Despite the growing evidence supporting the biogenesis and secretion of EVs, the mechanisms involved are still not fully understood. It is essential to continue studying the fundamental molecular mechanisms governing EV synthesis and secretion.^33^

### Components of extracellular vesicles

EVs contain lipids, nucleic acids, and proteins derived from their parental cells, and their composition can vary depending on the cell type or specific conditions.^43^ The molecular contents of EVs are influenced by the subtype of EVs, with larger EVs carrying more DNA, CD9, or Annexin A1, while smaller EVs are enriched with CD63 and CD81.^44^ The presence of DNA in EVs has garnered increasing attention. These DNA components encompass various forms, including but not limited to double-stranded DNA, single-stranded DNA, mitochondrial DNA, and even circular DNA forms such as extrachromosomal circular DNA (eccDNA).^45,46^ Recent studies suggest that DNA carried by EVs can reflect the genomic content of the cells of origin and, intriguingly, has been implicated in horizontal gene transfer between cells, contributing to genetic diversity and potentially enabling the spread of oncogenes during cancer progression.^47^ Moreover, DNA within EVs has been studied in the context of disease biomarkers. For instance, tumor-derived EVs have been found to contain tumor-specific genetic alterations, such as mutations or amplifications, making them a focus of intense research for non-invasive diagnostic approaches, including liquid biopsies.^48^ Furthermore, mitochondrial DNA in EVs has been linked to cellular stress responses and may play a role in signaling in the immune system.^49^ Given these multifaceted roles and the potential of DNA in EVs, its comprehensive analysis is critical, both for improving our understanding of EVs’ physiological roles and for harnessing their capabilities for diagnostic and therapeutic applications. Among the nucleic acids present in EVs, various forms of RNA are found, and they can undergo horizontal transfer between cells, leading to phenotypic changes in recipient cells.^50^ Different types of RNA, including mRNA, miRNAs, long noncoding RNA (lncRNAs), piwi-interacting RNA (piRNA), and circular RNA (circRNAs), have been identified in EVs, with miRNAs receiving significant attention due to their crucial roles in gene regulation and involvement in various physiological processes.^51^ MiRNAs are the most abundant RNA species in human plasma EVs, comprising approximately 40% of all sequencing reads in RNA sequencing analysis.^50^ Functioning lncRNAs and circRNAs have also been reported in EVs and can impact diverse biological processes, including cancer progression.^52^ EVs are enriched with various types of lipids on their surface, including ceramide, cholesterol, sphingomyelin, phosphatidylserine, and saturated fatty acids. Ceramide is the most abundant lipid responsible for EV formation.^44^ The lipid composition of EVs is characterized by decreased proportions of phosphatidylcholine and diacylglycerol and increased proportions of sphingomyelin, gangliosides, di-saturated lipids,^53^ and cholesterol^54^ compared to the parent cell. Lipid-enriched EVs can stimulate cell signaling pathways associated with cancer phenotypes,^55^ and phosphatidylserine lipids have been identified as cancer detection biomarkers.^44^ Proteins are another essential cargo of EVs, and their composition depends on the activity of the associated cell types.^56^ EVs contain proteins classified as cell membrane or cell membrane-associated, cellular, extracellular matrix (ECM), and serum proteins.^57^ Examples of proteins found in EVs include tetraspanins (CD9, CD63, CD81, and CD82), MVB-related proteins (TSG101, ALIX, and Rab proteins), heat shock proteins (HSP90 and HSP70), growth factors and cytokines (TNF-α, VEGF, EGF, TNF receptors, and TGF-β), cell adhesion-related proteins (integrins and intercellular adhesion molecule 1), antigen presentation-related proteins (major histocompatibility complex class I and II/peptide complexes), signaling proteins (GTPase HRas, Ras-related protein, Src, and RhoA), cytoskeleton components (actins, cofilin-1, moesin, myosin, tubulins, and vimentin), transcription and protein synthesis-related proteins (histone, ribosomal proteins, and ubiquitin), metabolic enzymes (fatty acid synthase, phosphoglycerate kinase, ATPase, and aldehyde reductase), death receptors (FasL and TNF-related apoptosis-inducing ligand), and iron transport proteins (transferrin receptor). These proteins can have functional effects on recipient cells, and their localization in EVs is important for their interaction with recipient cells.^44^ In addition, membrane proteins present in EVs can serve as disease biomarkers, as they can carry unique proteins that reflect specific patient conditions. These bioactive molecules have the potential to influence surrounding cells and can be targeted for therapeutic and biomarker applications. Notably, EVs carry high levels of program death-ligand 1 (PD-L1) and oncogenic receptors, contributing to disease progression, angiogenesis, and tumor growth.^58,59^ Therefore, analyzing EVs and their molecular contents, including lipids, nucleic acids, and proteins, holds great promise for understanding disease mechanisms, developing diagnostic tools, and exploring therapeutic targets.

## Sources of extracellular vesicles

### Extracellular vesicles in human body fluids

EVs are released from various cells in different tissues and biofluids and can be found in body fluids (Fig. 2). The origin of EVs provides valuable information about the heterogeneity of the tissue and cellular sources of circulating EVs. Interestingly, a study examined 101 samples of human plasma and found that 99.8% of circulating EVs are generated from hematopoietic cells, while only 0.2% originate from cells of other tissues.^60^ The exLR (extracellular RNA) profile was used to develop an EV-origin approach, which involved several steps such as processing tissue/cellular RNA-seq data, constructing and optimizing signature matrices, selecting and evaluating models, and exploring the atlas of EV origins from normal or disease samples using an identified algorithm.^61^

In the circulatory system, most EVs originate from platelets.^61^ Platelets, as key components of the blood clotting cascade, possess a variety of granules. Upon specific stimuli, these granules are released through a process involving the complement system, leading to the formation of MVs.^62^ This intricate mechanism underscores the pivotal role of platelets in not only hemostasis but also in the broader context of cellular communication and response within the bloodstream. Several cancer cell types, including glioblastoma (GBM), gastric cancer (GC), lung cancer (LC), and skin cancer (SC), are prolific producers of EVs.^2,63^ The interplay between platelet-derived EVs and their molecular cargo with components of the TME can wield a multifaceted influence. They can potentially augment cancer progression, restructure the TME landscape, and bolster metastatic endeavors.^64^ The circulatory reservoir of EVs is not solely platelet-centric. A plethora of immune cells, encompassing monocytes, macrophages, dendritic cells, natural killer (NK) cells, B and T lymphocytes, megakaryocytes, and endothelial cells, also contribute to the blood’s EV profile.^65–67^ Conversely, some tissues, such as adipocytes, muscle tissue, and cardiomyocytes, are relatively conservative EV producers.^68,69^ Intriguingly, cancer cells are adept at liberally discharging EVs not only into the bloodstream but also into tissue fluids. This characteristic amplifies the diagnostic potential of EVs, positioning them as valuable markers for the early detection and surveillance of various malignancies and other pathological conditions (Fig. 2). An in-depth exploration into the tissue-specific origins and heterogeneity of EVs can unravel a wealth of information, potentially revolutionizing our understanding of cellular diversity and offering a vanguard in diagnostic precision for myriad diseases.

### Stem cells derived extracellular vesicles

Stem cells have the potential to differentiate into various cell types in the body and play a crucial role in the body’s repair processes. There are two main types of stem cells: embryonic stem cells (ESCs) and adult stem cells. ESCs are pluripotent stem cells derived from the inner cell mass of a blastocyst, capable of self-renewal and differentiation into any cell type. EVs released by stem cells play a role in maintaining the survival and pluripotency of these cells.^68^ Specifically, a subtype of EVs called MVs produced by ESCs promotes trophoblast migration and facilitates blastocyst implantation into the uterine wall.^70^ Fibronectin on the plasma membrane of MVs activates focal adhesion kinase (FAK) activity within ESCs, contributing to maintaining their stem cell characteristics.^71^ Additionally, ESC-derived EVs impact retinal cells, such as in retinal degeneration (RD). The heat shock protein HSP90 present in ESC-derived EVs mitigates RD by facilitating retro-differentiation of retinal cells through upregulation of Oct4 expression.^72^ EVs derived from cancer stem cells (CSCs) are critical in cancer pathophysiology. They promote non-CSCs to acquire stem-like characteristics, exhibit chemotherapy resistance, and facilitate metastasis, angiogenesis, and immunosuppression.^73,74^ Like other cell types, adult stem cells, specifically mesenchymal stem cells (MSCs), also produce EVs. Previously, secretion by MSCs was believed to be limited to small molecules such as growth factors, chemokines, and cytokines.^75^ However, it has been demonstrated that MSCs secrete EVs in response to various chemical, environmental, and mechanical stimuli.^76^ MSC-derived EVs carry MSC-specific markers such as CD105, CD90, CD29, CD73, CD44, and KIT (CD117).^77^ These EVs can modify other cell types in the local or distant environment.^77^ MSC-derived EVs have been shown to promote the proliferation of primary CD34+ cells and hematopoietic stem cells (HSCs) derived from umbilical cord blood.^78^ They also prevent the apoptosis of HSCs and influence the fate of the hematopoietic system. On the other hand, MSC-derived EVs inhibit B-lymphocyte proliferation^79^ and display immunosuppressive effects by suppressing natural killer (NK) cell activity and interferon gamma (IFN-γ) production.^80^ In summary, EVs released by stem cells, including ESCs and MSCs, have diverse functions and can influence the behavior and characteristics of recipient cells in various contexts, including embryonic development, tissue repair, cancer progression, and immune modulation.

### Immune cell-derived extracellular vesicles

Immune cells, as crucial components of the body’s defense mechanism, are prolific producers of EVs. These immune cell-derived EVs play instrumental roles in modulating immune responses, be it activation, suppression, or communication.^81^ Almost all immune cell types, including T cells, B cells, dendritic cells, macrophages, and neutrophils, have been shown to produce EVs.^82^The process of biogenesis varies, with exosomes originating from endosomal compartments known as MVBs, while MVs form directly from the plasma membrane. Immune cells might increase their release of EVs in response to specific stimuli, such as during activation, differentiation, or under stress conditions.^82^ Immune cell-derived EVs carry a cargo that reflects their cell of origin and its functional state. This cargo can include proteins, lipids, miRNAs, and other bioactive molecules. For instance, EVs from dendritic cells might carry MHC-peptide complexes essential for T-cell activation. Likewise, EVs from T cells can have signaling molecules that modulate the activity of recipient cells. Immune cell-derived EVs play instrumental roles in modulating immune responses. They are involved in both adaptive and innate immunity, facilitating communication between immune cells and influencing their activation, differentiation, and effector functions.^82,83^ For instance, antigen-presenting cell-derived EVs can harbor major histocompatibility complex (MHC) molecules, presenting antigens to T cells and thus modulating adaptive immune responses.^84^ Interestingly, certain immune cell-derived EVs exhibit immunosuppressive properties. For example, regulatory T cell-derived EVs (Treg-EVs) have been shown to carry immunosuppressive molecules like CTLA-4 and TGF-β, contributing to maintaining immune tolerance and preventing autoimmune reactions.^85,86^ The involvement of immune cell-derived EVs in inflammatory processes is also notable. EVs released by neutrophils, macrophages, and other immune cells contain bioactive molecules (e.g., cytokines, chemokines, and lipid mediators) that can promote or resolve inflammatory reactions, implicating them in the pathophysiology of various diseases, from autoimmune disorders to cancer.^87^ The unique characteristics of immune cell-derived EVs have been exploited for therapeutic purposes. Their ability to present antigens has been harnessed in vaccine development, where they can enhance antigen presentation to immune cells, thereby boosting immune responses against targeted pathogens or tumor antigens.^88,89^ In conclusion, immune cell-derived EVs are pivotal players in orchestrating and modulation of immune responses. Understanding their biogenesis, cargo, and functional roles can provide insights into immune system function and offer novel therapeutic avenues. As research progresses, these tiny vesicles might become central players in both diagnostics and therapeutics in the realm of immunology.

### Pathogen derived extracellular vesicles

In recent years, the scientific community has witnessed an escalating interest in the EVs secreted by pathogenic entities, including bacteria and viruses. These minute vesicles are more than mere cellular by-products; they serve as vital tools for these microorganisms, facilitating a plethora of functions crucial for their survival and pathogenesis. Pathogen-derived EVs are laden with an array of biological molecules, encompassing nucleic acids, proteins, lipids, metabolites, and even virulence factors. These constituents play pivotal roles in various processes, including *Horizontal Gene Transfer*: Facilitating the exchange of genetic material between organisms, leading to rapid adaptation and evolution, *Cross-Kingdom Communication:* Enabling microorganisms to interact with hosts from different biological kingdoms, *Regulation of Host Immunity:* Modulating the host’s immune responses to favor pathogen survival and proliferation.

Both gram-negative and gram-positive bacteria have been identified as active secretors of EVs. Intriguingly, the secretion of MVs isn’t a novel or unique phenomenon. It’s a universally observed process, transcending the complexity of organisms, from the most rudimentary single-celled entities to intricate multicellular life forms.^90^ In gram-negative bacteria, the biogenesis of EVs involves the outward budding of their outer membrane. The resulting vesicles, ranging in size from 20 to 250 nm, often encapsulate components from the periplasmic space within their lumen.^91^ While much has been discovered, the precise molecular and mechanical pathways guiding vesicle formation in gram-negative bacteria remain active research areas.The biogenesis of EVs in gram-positive bacteria is distinct from their gram-negative counterparts. Their EVs predominantly arise from the cytoplasmic membrane, with the membrane and the vesicle’s lumen being sourced from the cytoplasm.^92^ An intriguing mechanism known as endolysin-triggered bubbling cell death is behind the formation of cytoplasmic membrane vesicles (CMVs) in these bacteria.^93^

Bacterial EVs carry various cargo molecules that can impact animal, plant, and bacterial cell biological processes. They play a role in mediating stress responses, biofilm formation, and influencing host cells. EVs are crucial for intra-species cell-to-cell communication, quorum sensing, and trafficking of nucleic acids, proteins, pathogen-associated molecular patterns (MAMPs/PAMPs), hydrophobic compounds, and horizontal transmission of antibiotic resistance between bacteria and hosts.^93^ Bacterial EVs specifically deliver toxins, pathogenic factors, and virulence factors to eukaryotic target cells. For example, Bacteroides fragilis, a member of the human microbiota, releases polysaccharide A capsular antigen (PSA) in outer membrane vesicles (OMVs). PSA from OMVs stimulates Toll-like receptor 2 (TLR2), leading to the production of Gadd45 in dendritic cells (DCs) and resulting in the production of the immunoregulatory cytokine IL-10, which promotes regulatory T cell (Treg) development.^94^

Bacterial EVs containing microbial-associated molecular patterns (MAMPs) can interact with immune and non-immune cells, including epithelial cells on mucosal surfaces. This interaction can affect host diseases, including inducing immune tolerance or conferring protective immunity. The specific impact of bacterial EVs depends on the particular parental bacterium and its relationship with the host.^95^ Several studies have demonstrated that patients with conditions such as intestinal mucositis, inflammatory bowel disease, or HIV exhibit elevated levels of circulating bacterial EVs carrying lipopolysaccharide (LPS) compared to healthy individuals. Furthermore, the integration of bacterial DNA sequences through OMVs has been observed more frequently in human cancer cells, particularly in tumors related to the gastrointestinal tract. This suggests that OMVs containing bacterial DNA may have a role in cancer development.^95^ The precise mechanisms by which bacterial EVs influence oncogenesis and tumor growth are not yet fully understood and are likely to be complex and context-dependent. However, research efforts are underway to utilize genetic engineering techniques to modify bacteria and isolate recombinant EVs for potential use as cancer vaccines.

EVs, such as MVs and exosomes, have emerged as pivotal players in viral propagation, providing a shielded conduit for both enveloped and non-enveloped viruses. By leveraging these vehicles, viruses can manipulate host responses, enhance their spread, and evade the immune system.^96^ EVs don’t just passively assist viruses; they actively regulate the infection process. For instance, EVs can enable viruses to exit host cells non-lytically, preserving the infected cell while establishing infections in new target cells.^97^ Several viruses, including HCMV, HHV-6, SARS-CoV-2, DV, HBV, HAV, HEV, EV71, and Bluetongue virus, exploit EVs as stealth devices. These vesicles conceal the viruses from immune surveillance, providing a cover that enhances viral transmission. As masters of adaptation, viruses have developed strategies to commandeer the host’s EV biosynthesis machinery during various phases of their life cycle.RNA and DNA viruses utilize components such as Rab-GTPases and the ESCRT complex to govern EV secretion, thereby furthering their spread.^98^ For example, the large hepatitis B surface proteins (LHBs) of HBV co-opt Rab5B, directing the virus towards EVs.^99^ Non-enveloped viruses, on the other hand, manipulate the ESCRT complex to envelop their virions or viral DNA within EVs.^98^ Hepatitis A (HAV), a hepatovirus, was first identified as being enclosed in exosome-like membrane vesicles formed from the host, shielding the virions from antibody-mediated neutralization. These viruses are infectious and are found to be present in the bloodstream of infected individuals, and they employ host ESCRT machinery for biogenesis. Another picornavirus, the aphtho virus that causes foot-and-mouth disease (FMDV), was discovered to be released from cells by an exocytic process involving membrane-limited vesicles.^100^ It has been shown that viruses effectively infect host cells through EVs, which increases their ability to propagate and circumvents the host’s defensive response. Recently, an exosome-mediated method for FMDV transmission has been reported both in vivo and in vitro, and it has been suggested as a possible means of immune evasion. Hepatitis E Virus (HEV) leverages the cellular exosomal pathway, getting released through MVBs, which then circulate in the blood encased in protective membranes during infection.^101^ The EVs containing HEV are just as infectious as the virus itself. Coxsackie B Virus (CBV)-infected cells produce a surge of EVs loaded with viral proteins and infectious virus.^102^ This process triggers mitochondrial fragmentation, allowing virions to be released within derived MVs. This “cloak” may help the virus evade the immune system, allowing efficient non-lytic viral spread. Human Polyomavirus 2 (JC) Virus, once inside the choroid plexus epithelial cells, encourages the production of vesicles containing virions. These vesicles then enter glial cells via pathways like macropinocytosis and clathrin-dependent endocytosis.^103^ Gastroenteric Non-enveloped pathogens like noroviruses and rotaviruses also utilize EVs for transport, boosting their fecal-oral transmission by delivering a concentrated infectious dose to subsequent host cells.^104^ In conclusion, pathogens, especially viruses, have ingeniously evolved to exploit the properties of EVs to their advantage. By hijacking and co-opting these vesicles, they not only ensure their survival but also complicate therapeutic interventions aimed at halting their spread. Understanding these interactions will be crucial in devising strategies to counteract viral transmission and pathogenicity.

### Isolation and identification of extracellular vesicles

EVs are actively secreted by mammalian cells and form a heterogeneous population. This heterogeneity poses a challenge when trying to detect specific subtypes of EVs, particularly in a population of normal and cancer cells. To overcome this, researchers commonly identify marker proteins such as CD91, CD317, and epidermal growth factor receptor (EGFR), expressed in most EVs. The detection and isolation of EVs have garnered significant interest in research due to their potential applications in identifying and isolating disease-related EVs, diagnosing multiple diseases at early stages, and identifying biomarkers of disease progression. Several methods are employed to detect and isolate EVs, primarily based on the expressed proteins and lipids. These methods are summarized in (Table 2).

**Table 2:** Techniques used for detection and isolation of extracellular vesicles

S. No.	Techniques for detection and isolation of EVs	Sub-types of detection and isolation techniques of EVs	Advantages	Disadvantages	Refs.
1	Filtration-based techniques for EV isolation.	Centrifugal ultrafiltration, Tangential flow filtration, Exodisc, ExoTIC (exosome total isolation chip), Integrated double-filtration microfluidic device, Hydrostatic dialysis	High purity, Fast, Scalable, Simple	Time-consuming, Low purity, lower EV yield, reduced sample recovery, Protein contamination	^606–612^
2	Flow field-flow fractionation-based techniques	Immunoaffinity chromatography - asymmetrical flow field-flow fractionation (IAC-AsFlFFF), Frit-inlet AsFlFFF	Avoiding yield loss, reducing potential EV’s integrity damage, and Scalable	The AF4 process requires high expertize to operate and customize; tangential flow filtration is often not used as an EV purification method	^606,613–616^
3	Size-based/ Density-Based /Centrifugation technique	Size-exclusion chromatography, Differential ultracentrifugation, CUC: cushioned-density (ultra)centrifugation, DGUC: density gradient (ultra)centrifugation, Sucrose density gradient centrifuge, Iodixanol density gradient ultracentrifugation	Low cost, Low risk of pollution Fast, Scalable, Simple, Easily automated, and integrated with diagnosis	Protein contamination; sample volume limited, Low extraction volume; Extensive laboratory equipment requirements Time-consuming, operator and equipment-sensitive process, purity depending on the optimization based on starting sample type, rotor used, and the applied g-forces	^606,617–620^
4	Ion-exchange based techniques	Anion-exchange chromatography, Anion exchange, Nickel-based isolation, Cation- and anion-exchange chromatography	Require shorter isolation time, higher purity	Ion-exchange methods in EV research are limited to cell culture but face challenges in complex biological matrices like blood and plasma due to high amounts of charged biomolecules	^621–624^
5	Electrophoresis and dielectrophoresis (DEP) based techniques	Alternating current electrokinetic, microarray chip device, Agarose gel electrophoresis, Capillary electrophoresis, Capillary zone electrophoresis, Direct current–insulator-based dielectrophoresis, Electrophoresis with dialysis, On-chip immunoelectrophoresis, On-chip microcapillary electrophoresis		The electric field has the potential to influence the properties of exosomes	^625–627^
6	Affinity-based EV isolation and separation techniques.	Magnetic beads, Silica nano spring, Agarose resin, Polymeric monolithic disks, Agarose gel column, Immunoaffinity Enrichment, Immunocapture, Enzyme-Linked Immunosorbent Assay (ELISA)	High purity to isolate specific EVs subtypes	The affinity approach to EV removal is limited by factors like beads’ binding capacity, antigen exposure, epitope stability, antibody affinity, acidic elution buffers, and high costs	^606,628,629^
7	Methods utilizing the change in EV solubility and aggregation	Precipitation with Hydrophilic Polymers, Precipitation with Protamine, EV Precipitation with Sodium Acetate, Precipitation of Proteins with Organic Solvent (PROSPR)	The process is quick, easy, and scalable; it doesn’t damage electric vehicles (EVs), and it doesn’t need any special isolation equipment.	The sample may be contaminated with proteins, complexes, lipoproteins, nucleoproteins, viral particles, and biopolymers, potentially affecting further analysis, long process, gel filtration is required, PROSPR technique is inferior to gel chromatography, acetone can disrupt the functionality of vesicular membranes	^630–632^
8	EV isolation methods utilizing interactions	Antibodies to EVs receptors, Phosphatidylserine-Binding proteins, Heparin modified sorbents, Binding of heat shock proteins, lectins.	Low cost, simple, high purity, preservation of functional integrity, readily reversible bonding, does not require complex equipment.	Obstacles include detachment, intact vesicle analysis, nonspecific binding, initial purification and concentration requirements, high selectivity, cost, and antibody availability	^632–638^
9	Microfluidics	Microfluidics-based immunoaffinity capture, acoustofluidics, membrane-filtration microfluidics, viscoelastic flows or nanowire traps, Viscoelasticity-based microfluidic system, λ-DNA mediated viscoelastic microfluidic system, Electroosmotic flow-driven DLD pillar array.	Low cost, Fast, Simple, Easily automated and integrated with a diagnosis	Requires a specific level of expertize, not suitable for preparative purposes (e.g., therapeutic applications), low sample volume might be a limitation, need additional equipment, high cost	^606,639,640^
10	Precipitation based isolation	Commercial kits for polymer precipitation, polymer precipitates EV, Urine Exosome RNA Isolation Kit, Total Exosome Isolation Solution, and RIBO™ Exosome Isolation Reagent	User-friendly, cheap, simple, and does not require complex equipment.	Costly for high sample sizes, post-cleanup is required for downstream applications due to polymer and protein contamination.	^606,641^
11	Other techniques used for the detection and isolation of EVs	nano-sized deterministic lateral displacement, Oscillatory viscoelastic, NanoDLD pillar array, Electroosmotic flow-driven DLD pillar array, nanoplasmon-enhanced scattering assay, ExoSearch Chip, Acoustic Nanofilter, Facile PEG-based isolation, Nanoparticle tracking analysis, Contact-Free Sorting, Two-phase isolation, KeepEX, Label-Free SERS to Identify EV, using carboxyl group-functionalized iron oxide nanoparticles, Matrix-assisted laser desorption ionization time-of-flight mass spectrometry	Higher yield, DLD is a nondestructive method that enables rapid, continuous, single-particle sorting in a continuous flow, without particle labeling, using small sample volumes.	Need more equipments, a lengthy process, Limitation on the number of samples that may be processed simultaneously (to six samples).	^642–652^

### Role of extracellular vesicles in cancer biology

**Fig. 3:**
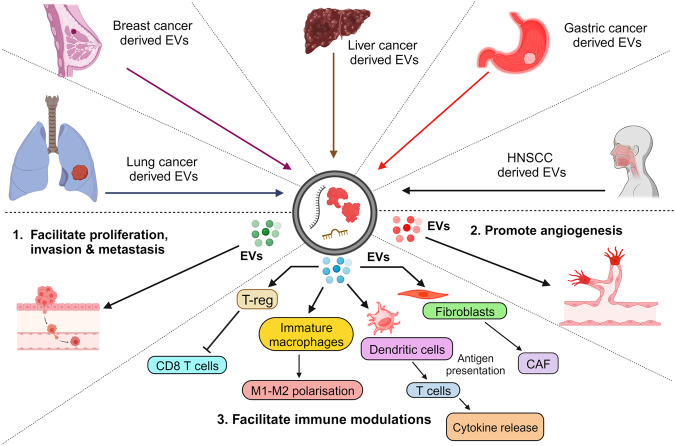
Comprehensive role of extracellular vesicles in cancer progression. This diagram delineates the intricate interplay of EVs in various cancer dynamics. Through the transportation of specific EV-associated molecules, they govern a range of tumorigenic processes, including: 1. Invasion and Metastasis: The cargo within EVs can promote the breakdown of extracellular matrix, paving the way for cancer cells to invade surrounding tissues. EVs can impart migratory capabilities to tumor cells, aiding their movement and potential metastatic spread. 2. Angiogenesis: By transmitting pro-angiogenic factors, EVs form new blood vessels, thereby supporting tumor growth and expansion. 3. Immunomodulation: EVs can modulate the TME by influencing the behavior of immune cells, potentially facilitating tumor evasion from immune surveillance

EV-related pathways have been extensively analyzed in cancer cells since the initiation of EV research.^105^ Substantial data support the hypothesis that EVs released from tumors and the surrounding cells play crucial roles in cancer biology (Fig. 3).^56^ In the context of cancer, EVs can promote the formation of supportive TME and (pre)metastatic niches, facilitating the establishment and propagation of tumor cells.^17,106^ Active communication between tumor cells, neighboring cells, and the local microenvironment is necessary when a tumor develops at the primary site. The utilization of EVs by cancer cells to establish an optimal TME for disease progression has attracted significant interest.^17^ During cancer initiation, a conflict exists between newly transformed cells and surrounding epithelial cells. Non-cancerous cells actively release growth-inhibitory miRNAs to eliminate transformed cells and prevent tumor initiation.^107^ In cancer cells, the expression of tumor-suppressive miRNAs is downregulated.^108^

Consequently, the continuous provision of tumor-suppressive miRNAs via EVs represents a homeostatic mechanism that tumor cells must overcome. Once this balance is compromised, the microenvironment becomes susceptible to tumor initiation. EVs derived from cancer cells have been implicated in determining the tumorigenic potential of normal cells. For instance, EVs derived from prostate cancer cells and enriched in miR-424 have been proposed to induce stemness and tumorigenesis in normal epithelial cells.^109^ Tumor Derived EVs (TDEVs) also promote angiogenesis, disrupt vascular endothelial barriers, and can contribute to cancer metastasis. A study by Maji et al. demonstrated that metastatic BC-derived EVs, highly expressing Anx II, promote angiogenesis in an in vivo Matrigel plug assay.^110^ Two other studies reported that EVs secreted from metastatic BC cells disrupt the formation of tight junctions.^111^ Tumor EVs may also participate in epithelial-to-mesenchymal transition (EMT), a critical process in invasion, chemoresistance, and metastasis.^112^ For example, the overexpression of HRAS in Madin-Darby canine kidney epithelial cells promotes the packaging of mesenchymal markers (e.g., vimentin and MMPs) in exosomes, potentially inducing EMT in recipient cells.^113^ Immune escape is one of the hallmarks of cancer, and cancer cell-derived EVs involved in immunosuppression play essential roles in conferring advantages to cancer cells in evading attacks from immune cells.^114^ For instance, EVs can suppress natural killer (NK) cell and T-cell activity to enhance immune evasion.^44^ Yen et al. reported that EVs from cancer cells promote the expansion of regulatory T cells through TGF-β1 contained in the EVs.^115^ Hypoxic conditions also impact the function of EVs, and EVs derived from cancer cells under hypoxic conditions are enriched in miR-23a, resulting in the blockade of NK cell function.^116^ Cancer cells have been found to educate fibroblasts through EVs, leading to metastasis progression.^117^ This EV-mediated communication leads to the reprogramming of normal stromal fibroblasts into activated cancer-associated fibroblasts (CAFs) in various cancers such as chronic lymphocytic leukemia,^118^ hepatocellular carcinoma (HCC),^119^ and melanoma.^120^ These findings showed that cancer cells and fibroblasts engage in cross-talk via EVs to create a metastatic niche. Collectively, these findings on the function of EVs derived from cancer cells and stromal cells in metastasis provide new insights into the potential clinical application of EVs in treating cancer metastasis.

### Head and neck squamous cell carcinoma

HNSCC, the seventh most common global cancer, with over 900,000 new cases and 450,000 cancer-related deaths, occurred in 2020. By releasing EVs and developing a premetastatic TME, heterogeneous tumor cells can enhance the motility and angiogenic activity of neighboring tumor cells. Uncertainty exists about the active chemicals that control tumor growth in HNSCC-derived EVs. Proteomic studies showed that several tumor-associated proteins, including TRAP1, EGFR, HSP-90, and MMP-13, are present in EVs produced from HNSCC. Evidence from both in vitro and in vivo studies indicates that TGF, which HNSCC-derived EV carries, significantly encourages tumor growth by promoting angiogenesis in the TME and immune evasion.^121^ Through various mechanisms, EVs carrying TGF stimulate tumor development and pro-tumor activity in the TME. TGF + EVS promoted macrophage chemotaxis without causing a major M1/M2 shift, converting primary human macrophages to a pro-angiogenic phenotype marked by elevated pro-angiogenic factors.^121^ EV-packaged TGFβ1 can reprogram normal fibroblasts into CAFs in vitro and in vivo by activating the TGFβ-Smad signal pathway and promoting cancer development.^122^ According to one study, TGF-1 in EV started NFs by controlling fibronectin instead of altering the traditional TGF-Smad signal pathway. These HNSCC-derived EVs differ from TGF-activated CAF in that they activate the pro-inflammatory gene IL-6 and the hypoxia-related genes NF-B, HIF1, HK2, and PFKL.^123^ There is growing evidence that miRNAs play a significant role in TGF-β signaling. TGF-1 has been shown to increase the expression of miR-21 in various cells. An oncogenic miRNA, miR-21, encourages both growth-promoting and anti-apoptotic capabilities. HNSCC is a common multifactorial malignant tumor that arises from the epithelial lining of the oral and nasal cavities, larynx, and pharynx.^124^ A study identified 108 miRNAs derived from EVs of HNSCC, among which miR-21 and HOX transcript antisense RNA (HOTAIR) were found to be significantly upregulated under hypoxic conditions. MiR-21 was associated with OSCC (Oral Squamous Cell Carcinoma) cell migration, invasion, T-stage, and lymph node metastasis in OSCC patients. It achieves this by upregulating snail and vimentin expression while downregulating E-cadherin levels.^125^ In nasopharyngeal carcinoma (NPC), miR-23a is secreted by tumor cells and transported to epithelial cells via EVs. It targets and downregulates the tumor suppressor iR-TSGA10. Decreased expression of miR-TSGA10 promotes angiogenesis and metastatic progression.^126^ Similarly, miR-494 and miR-142-3p, secreted by OSCC tumors, promote metastasis and angiogenesis by activating the NOS and TGFBR1 pathways in endothelial cells.^127,128^ Other miRNAs abundant in EVs derived from NPC tumors include hsa-miR-24-3p, hsa-miR-891a, hsa-miR-106a-5p, hsa-miR-20a-5p, and hsa-miR-1908. These miRNAs downregulate the mitogen-activated protein kinase (MAPK) signaling pathway, leading to T-cell dysfunction, impaired proliferation, differentiation, and tumor immune evasion.^129^ Furthermore, miR-34a-5p derived from CAFs promotes the progression of OSCC through the AKT/GSK-3β/β-catenin signaling cascade.^130^

### Gastric cancer

Gastric cancer (GC) is the fifth-leading and one of the deadliest types of cancer worldwide. Emerging research has shown the association of EVs with GC.^131^ Studies examining EVs from GC patients and cell lines have identified differential expression of GC-associated proteins and RNAs. For instance, MAGE-1 and HER-2/neu mRNA were significantly overexpressed in EVs from five patients with stage IV GC.^132^ Abundant expression of miR-21, miR-30a, miR-1290, and miR-1246 has been found in EVs derived from GC stem-like cells.^133,134^ Deep sequencing of RNAs from a cohort of GC patients revealed high expression of miR-217, which negatively impacts CDH1 levels and subsequent cancer cell proliferation.^135^ LncRNA ZFAS1 and miR-423-5p were upregulated in GC cells, tumor tissues, and serum in GC patients’ EVs. These molecules were associated with lymphatic metastasis and the TNM stage.^136^ Communication between TDEVs, normal cells, and the TME is critical in tumor metastasis. High expression of CD44v6, TGF-β1, and CD97 plays a central role in forming premetastatic niches in the GC and regional lymph nodes.^137–139^ EGFR derived from GC cells and integrin αvβ5 EVs has been associated with liver metastasis.^140^ EGFR, when transported via EVs to the liver, promotes liver-specific metastasis in GC through liver miR-26a/b and HGF signaling.^140^ By delivering c-Myc, TDEVS increased GC cell proliferation, invasion, and migration. TDEVs expressing c-Myc promote GC cell proliferation, invasion, and migration by protecting the disruption of the PI3K/AKT pathway by blocking miR-556-3p expression. c-Myc upregulates KCNQ1OT1 to elevate CLIC1 expression, thereby activating the PI3K/AKT pathway.^106^ Particularly, TDEVS containing c-Myc suppressed miR-556-3p expression via upregulating KCNQ1OT1 to increase CLIC1 expression, activating the PI3K/AKT pathway and accelerating GC cell proliferation, invasion, and migration.^141^ MiR-130a, another miR, promotes angiogenesis in GC through interacting with C-MYB in vascular endothelial cells in vitro and in vivo.^131^ MiR-130a targets the 3’-UTR C-MYB mRNA in HUVECs, which reduces the expression of the CMYB protein, increasing angiogenesis and tumor development. TDEs can create an immunosuppressive environment by attenuating the immune response and recruiting immunosuppressive cells. A recent study suggested that TDEs carrying programmed cell death 1 ligand 1 (PD-L1) retain immunosuppressive activity by downregulating T-cell surface CD69.^142^ Recent research has shown that EVs produced by tumor cells also exhibit significant PD-L1 (Programmed Death Ligand) expression levels. PD-L1 greatly influences immunosuppression and immunotherapy resistance on EVs, which can attach directly to PD-1 on T cells. PD-L1 expression in donor cells was found to correlate with EV PD-L1 secretion in human GC cell lines. They also found that a higher baseline circulating EV PD-L1 level was linked to poorer overall survival in 31 metastatic GC patients following chemotherapy.^143,144^ An in vitro experimental model revealed that GC-derived EVs control neutrophils in the GC to mediate immunosuppression via HMGB1. HMGB1 induces PDL1 expression in neutrophils through activation of the STAT3 signaling pathway. The PD-L1/PD-1 interaction causes the PD-L1+ neutrophils to decrease T cell activity, which has a pro-tumor impact.^145^

TDEVs miR-21-5p promotes GC peritoneal metastasis by inducing EMT and targeting SMAD7.^146^ Under pathological conditions, EVs carrying miR-15b-3p enhance tumorigenesis and malignant transformation by suppressing the NYDLT1/Caspase-3/Caspase-9 pathway and apoptosis in GC.^147^ EVs also promote the growth and metastasis of GC by transporting N-recognin 2 (UBR2), a component of the ubiquitin protein ligase E3, to GC cells. UBR2 activates the Erk/MAPK pathway by inhibiting the negative regulator of the pathway through ubiquitination.^148^ Lymphatic metastasis, a standard route of metastasis in GC, is caused by the miR-877-3p/VEGFA and SPRY4-IT1/miR-101-3p/AMPK axes mediated by overexpression of circRanGAP1 and lncRNA SPRY4-IT1, respectively.^149,150^ These molecules, circRanGAP1, and SPRY4-IT1, are closely associated with the progression, lymphatic metastasis, and poor survival in GC. Under hypoxic conditions, overexpression of lncRNA PCGEM1 in EVs reduced the degradation of SNAI1 in GC, inducing the invasion and metastasis of GC cells.^151^ An in vitro study demonstrated that EVs derived from highly metastatic diffuse GC cells and carrying miR-193b induce chemokine production in fibroblasts known as CAFs. This effect is achieved through the upregulation of CXCL1 and CXCL8 expression. The presence of these EVs and the subsequent chemokine production have been closely associated with a poor prognosis and the progression of GC.^152^

### Lung cancer

Lung cancer (LC) is responsible for the highest number of cancer-related deaths globally, accounting for nearly 25% of all cancer deaths.^153^ There are two main types of lung cancer: non-small cell lung cancer (NSCLC) and small cell lung cancer. The cargo carried by lung cancer-derived EVs (LCEVS) interacts with the TME, playing a role in tumor initiation and progression and serving as potential non-invasive biomarkers for cancer diagnosis. EVs derived from LC cells containing miR-21 and miR-29a bind to and activate toll-like receptor 8 (TLR8) on immune cells within the TME. This activation of TLR8 triggers NF-κB expression, which leads to pro-metastatic inflammatory responses, LC progression, and metastasis.^154^ Studies have demonstrated that LCEVS, under hypoxic conditions, contains miR-619-5p, which promotes angiogenesis by suppressing RCAN1.4.^155^ TDEVs have been shown to establish an immune-suppressive environment by polarizing macrophages in LC. They activate TLR2 on macrophages, increasing the expression of PD-L1 through metabolic reprogramming and the NF-kB transcription factor, utilizing HIF-1a/GLUT-1.^156^ During hypoxic conditions, LCEVS carry elevated transforming growth factor (TGF)-β, EGFR, and miR-23a, creating an immunosuppressive tumor-associated macrophage (TAM) population. EVs play a crucial role in the onset and progression of NSCLC, with over 50% of EVs from NSCLC cell lines staining positively for EGFR. Studies have found that EVs from NSCLC include mutant versions of EGFR, which promote proliferative signaling, invasion, and cancer cell metastasis in tumor tissue of NSCLC patients.^157^ EGFR also promotes angiogenesis through EGFR-dependent autocrine VEGF expression in endothelial cells.^158^ MiR-23a indirectly causes the degradation of NK cells by targeting CD107a,^159^ while EGFR suppresses CD8 + T cells by inducing tumor antigen-specific regulatory T cells (Tregs).^160^ EGFR is indirectly activated by LC-derived miR-494-3p, which targets PTPN12, a negative regulator of EGFR and VEGFR2 receptor tyrosine kinases.^161^ Oncogenes H-Ras and K-Ras in cancer cells mediate the expression and release of miR-23a through syntenin-1 in EVs.^161^ Mutations in the RAS,^162^ p53, and PI3K pathways,^163^ altered EGFR,^164^ as well as microenvironmental factors such as tumor-associated fibroblasts (TAF), have been identified as significant mediators of LC progression, recurrence, and metastasis.^165^ LCEVS with elevated levels of vimentin induces EMT in human bronchial epithelial cells.^160^ EMT causes protein changes in EVs, detectable in NSCLC patients, and induces chemoresistance and metastatic potential in recipient LC cells. Early tumor formation is typically marked by hypoxia, leading to an aggressive, resistant-to-treatment, invasive, and metastatic phenotype. It is shown that EMT leads to increased levels of the EV hypoxic signature proteins. Previous research found that the six upregulated proteins GANAB, VCP, PSMA2, TNC, THBS1, and MAC2BP were all highly enhanced in hypoxic EVs made from NSCLC cell line sources. The EV signatures were cancer-specific and linked to EVs produced from NSCLC cells exposed to hypoxia.^166^ EVs have become crucial regulators of drug resistance, directly sequestering anti-tumor drugs and reducing their effective concentration in target areas. The P-gp gene, also known as ABCB1, is the primary drug transporter in MDR tumors and has been linked to tolerance to at least 20 chemotherapy drugs. EVs from resistant cells also included the copper-transporting P-type ATPases ATP7A and ATP7B and the multidrug resistance-associated protein 2 (MRP-2). ATP-binding cassettes (ABC) can localize to the limiting membranes of EV-like structures, promoting drug sequestration.^167^ The uptake of LCEVs induces pathogenic conditions in both normal and transformed recipient cells. Recent studies have shown that LCEVs carrying specific miRNAs induce invasion, modulate barriers in non-tumorigenic recipient cells, and mediate drug resistance.^168^ LCEVs carrying ALAHM, when delivered to the liver, promote the expression of ALAHM in liver cells, thereby facilitating liver metastasis of LC cells.^169^ ALAHM significantly activates the expression of HGF protein levels in liver tissue by binding to RNA-binding proteins. In summary, LCEVS and their cargo play crucial roles in LC progression, immune modulation, angiogenesis, metastasis, and establishing an immunosuppressive TME. Understanding these mechanisms can provide valuable insights into LC biology and potentially lead to developing novel diagnostic and therapeutic strategies.

### Breast cancer

Breast cancer (BC) is a prevalent malignancy among women worldwide, surpassing LC and ranking as the second most common cancer in the United States. Similar to other types of cancer, BC cell-derived EVs (BCEVs) are gaining attention as a valuable resource for detecting biomarkers and diagnosing early-stage disease. The cargo carried by BCEVs plays a critical role in the progression and metastasis of BC, as well as in the establishment and reprogramming of the local microenvironment and distant sites. Hypoxic tumors in various cancers are aggressive and lead to worse patient outcomes. EVs released in response to hypoxia-inducible factor-1 facilitate tumor development, angiogenesis, and metastasis. Hypoxia increased the expression of the RAB22A gene in advanced BC, subsequently enhancing BC invasion and EVs production.^170^ Analyzing BCEVs through molecular profiling offers a promising approach to gathering information about the parental cancer cells and the expression of disease-associated proteins and RNA within the EVs, serving as potential disease-specific biomarkers. Under hypoxic conditions, BCEVs activate the production and release of inflammatory cytokines and promote mitochondrial dynamics by activating the NFκB factor in recipient normal mammary epithelial cells. This activation of NFκB alters the physiology of both local and distant cells and microenvironments, contributing to tumorigenesis and metastasis.^171^ In response to pro-inflammatory cytokines, NF-κB has been shown to stimulate the migration and proliferation of human MSCs.^172^ Nuclear factor- kB (NF-κB) and the proinflammatory cytokines interferon-γ and tumor necrosis factor (TNF-α) jointly inhibit the self-renewal and differentiation of MSCs. More intriguingly, prolonged high levels of IFN-γ and TNF-β boost MSCs’ vulnerability to malignant transformation through NF-κB-mediated activation of c-Fos and c-Myc oncogenes.^172^ MSCs produce cytokine receptors and chemokines that interact with tumor-released chemicals, enabling them to integrate into the TME. MSC-EVs promoted the proliferation, migration, and invasion of BC cells via the activation of the ERK pathway. ERK can facilitate the differentiation of epithelial-like cells into interstitial cells, thereby inducing EMT and promoting cell migration and metastasis.^173^ Highly activated and upregulated IGF-1 released from BCEVs contributes to the development and progression of BC. BCEVs containing activated IGF-1 decrease E-cadherin levels, increase vimentin and N-cadherin expression, and stimulate the secretion of metalloproteinase-9 in mammary non-tumorigenic epithelial cells.^174^ These proteins are involved in EMT in mammary non-tumorigenic epithelial cells and play a crucial role in BC’s invasion and metastasis processes.^175^ BCEVs, which contain elevated levels of EDIL3, a metalloprotease protein, enhance the migration of less aggressive BC cell lines.^176,177^ EDIL3 induces cell invasion through the integrin-FAK signaling cascade in BC and promotes lung metastasis in vivo.^178^ BCEVs also carry VEGF90K, which is transferred to endothelial cells, leading to an upregulation of VEGF expression and activation of VEGFRs1. This process promotes tumor angiogenesis and influences the TME.^179^ Recent studies have identified differential expression of cancer-associated molecules in BCEVs, including HER2,^179^ EGFR,^180^ FAK, survivin, EMMPRIN, CD24, EpCAM,^181^ glypican-1 (GPC1), fibronectin,^182^ and developmental endothelial locus-1 (EDIL3),^181,183^ as well as specific miRNAs, when compared to healthy controls.^184^ BC cells became resistant to anoikis when EMMPRIN was expressed, which was accomplished by downregulating the pro-apoptotic BH3-only protein via a MAP kinase-dependent mechanism.^185^ Most breast tumors overexpress the survival protein, which confers resistance to chemotherapy and radiation. It has been discovered that the expression of HER 2 and EGFR is correlated with the overexpression of surviving.^186^ Furthermore, EVs derived from different cancer cells, such as CAFs, can influence BC. Normal fibroblasts are subjected to BCEVs, which cause them to develop a CAF-like phenotype. In particular, BC-derived EVs carrying miR-125b is transferred to fibroblasts, promoting a CAFs-like phenotype via activation of CAFs markers such as Acta2, MMP-2, and MMP-3. Similarly, transfer of miR-146a to fibroblasts activates the Wnt/β catenin pathway, which leads to the induction of a CAF phenotype.^187^ CAFs also secrete EVs that promote metastasis in BC. Suppression of certain miRNAs in EVs produced from CAF has been found to promote pro-tumorigenic characteristics in recipient cells. MiR-3188 is lost in CAF-derived exosomes, leading to the de-repression of B-cell lymphoma 2 (BCL2), accelerating tumor growth. Similarly, miR-7641, derived from CAFs, has been found to suppress BC cell stemness by regulating the HIF-1α pathway. CAF-derived cargo exhibits significantly lower levels of miR-7641 compared to other sources.^188^ CAF-derived cargo exhibits significantly lower levels of MiR-7641 compared to other sources. Analyzing BCEVs through molecular profiling offers a promising approach to gathering information about the parental cancer cells and the expression of disease-associated proteins and RNA within the EVs, serving as potential disease-specific biomarkers.

### Hepatocellular carcinoma

The role of EVs in liver disorders, such as alcoholic liver disease (ALD), viral hepatitis, and HCC, is increasingly being recognized.^189^ EVs facilitate communication between different cell types within the liver and between organs by carrying bioactive molecules. Liver cancer cells secrete more EVs than other cancer cells, which can have both pro-metastatic and anticancer effects, including the activation of natural killer cells for antitumor immunity.^190,191^ Heavy alcohol consumption leads to ALD, a multifactorial liver disease that can manifest in various clinical phenotypes, including HCC.^192^ Previous research on the functions of EV cargos in cancer has shown that EVs play a role in nearly all of the disease’s hallmarks, including tumor initiation and development, TME remodeling, apoptosis, angiogenesis, metastasis, immunological evasion, and treatment resistance. Like exosomal miR-21 from HCC, EVs can influence TME remodeling by converting hepatic stellate cells (HSCs) into CAFs and facilitating TME formation. Exosomal miR-1247-3p from HCC cells has been demonstrated to activate fibroblasts through the NF-κB signaling pathway by decreasing the expression of B4GALT3 in CAFs and stabilizing 1-integrin. ALD patients have elevated levels of circulating EVs in their serum. Alcohol-fed mice show increased levels of specific miRNAs such as miR-30a, miR-30b, miR-122, miR-192, miR-744, and miR-1246 in the cargo of EVs recovered from their serum compared to control mice. Similar high expression of miR-192 and miR-30a has been observed in human alcoholic hepatitis.^193^ Alcohol-induced liver injury triggers the release of more EVs, leading to apoptosis and disruption of lysosomal activity. EVs derived from alcohol-exposed monocytes exhibit high expression of miR-27a, promoting the polarization of naive monocytes into M2 macrophages.^194^ According to recent research, EVs produced by HCC can inhibit the activity of NK and T cells and stimulate immune suppressive cells. Heavy alcohol use is associated with a 68–87% increase in the risk of HCC, while light to moderate alcohol consumption is linked to a reduced risk.^195^ Recent research has shown that the exosome-enriched fraction of EVs can control hepatitis C (HCV) infection. Moreover, EVs can spread HCV infection to uninfected hepatoma cells, initiating viral replication in the newly infected cells. Patients with chronic HCV infection have higher levels of circulating serum EVs containing HCV RNA and elevated platelet activation and platelet-derived EVs in the blood.^196,197^ Substantial evidence implicates EVs in the development and metastasis of HCC. EVs derived from malignant HCC cells carry various oncogenic proteins and mRNAs, including the MET proto-oncogene. The uptake of these EVs by HCC cells can activate the PI3K/AKT and MAPK signaling pathways, leading to increased secretion of active MMPs and enhanced migratory and invasive abilities of hepatocytes.^198^ EV-derived Golgi membrane protein 1 (GOLM1) activates MMP-1 and MMP-9 in recipient cells.^198^ Oncogenic miRNA-carrying EVs, such as miR-93, miR-224, miR-665, miR-10b, and miR-21, promote HCC proliferation and metastasis.^199–201^ EVs derived from CAFs and TAMs also regulate HCC progression,^202^ with reduced levels of miR-125a/b in TAM-derived EVs suppressing HCC proliferation and stem cell characteristics.^203,204^ Furthermore, extracellular EVs derived from HCC promote tumor growth and control angiogenesis and new blood vessel formation.^205^ EVs released from HCC contain molecules such as miR-155, lncRNA-H19, and circRNA-100338, linked to angiogenesis.^206,207^ In HCC, the vascular endothelial growth factor (VEGF) protein directly stimulates the proliferation of hepatocytes, cancer cells, and epithelial cells, leading to abnormal vascular architecture. One specific miRNA, miR-32-5p, has been identified as the most highly expressed miRNA in EVs released from Bel/5-FU, and it has been shown to upregulate VEGF levels in vitro.^208^

### Biomarker potential of extracellular vesicles

EVs have emerged as essential players in cell-to-cell communication, normal cellular processes, and tumor development. They carry a cargo of proteins and miRNAs, making them potential biomarkers for diagnosing and prognosis of various diseases, including different types of cancer. Researchers are exploring using EVs and their cargo as tools for early cancer detection and therapy and monitoring treatment responses.^209^ Identifying specific EV proteins as novel diagnostic and prognostic biomarkers for LC is particularly promising. Increased expression of EGFR has been observed in LC cells, and higher levels of EGFR expression have been detected in EVs from LC patients compared to healthy controls.^210^ Studies analyzing patients with NSCLC have shown that elevated levels of exosomal miR-378 are associated with lymph node metastasis and advanced TNM stage, indicating its potential as a non-invasive prognostic biomarker.^211^ Furthermore, high expression of exosomal miR-146a-5p has been linked to cisplatin response, while miR-425-3p and miR-96 have been associated with cisplatin resistance in LC.^209^ Similar investigations have been conducted in HNSCC, where altered expression of miRNAs, such as miR-186, miR-3651, and miR-494, has been observed in whole blood samples from HNSCC patients compared to healthy individuals. Salivary EVs from OSCC patients have also shown significantly higher levels of specific miRNAs, including miR-21, miR-184, miR-412-3p, miR-512-3p, miR-27a-3p, miR-302b-3p, miR-517b-3p, and miR-494-3p, compared to healthy controls.^209,212–214^ Furthermore, high expression of miR-486-5p, miR-486-3p, and miR-10b-5p has been observed in HNSCC cell lines.^212,215^ The makeup of EV components differs significantly between HNSCC and normal cells. In a study involving HNSCC patients, specific biomarker candidates, including FAS, RET, STAT5, TNFRSF1B, WNT1, ABCB1, CASP5, CCND1, FGF1, ABL1, BCL2L1, PRIM1, CD4, HSP90AA1, and HSP90AB1, were detected in EVs from pre-treatment tumor tissues. In contrast, BAX, CASP3, HDAC1, NGFR, TNFSF11, TP73, BRCA2, EGFR, IKBKB, STAT1, SNAI1, BAG1, and TNFRSF10B were detected in EVs from patients who showed complete responses to treatment.^215,216^ Identifying specific EV proteins as novel diagnostic and prognostic biomarkers for LC is particularly promising. Plasma EVs from lung adenocarcinoma (LUAD) are observed to consistently have higher levels of Ras Homolog Family Member V (RHOV). Tetraspanin-8, CD171/L1CAM, and CD151/tetraspanin-24 were shown to be substantially expressed in histology lung tumors using an EV array.^217^ In BC, several phosphoproteins, including Ral GTPase-activating protein subunit alpha-2 (RALGAPA2), cGMP-dependent protein kinase 1 (PKG1), tight junction protein 2 (TJP2), and nuclear transcription factor X box-binding protein 1 (NFX1), are significantly upregulated.^218^ Additionally, the levels of epithelial cell adhesion molecule (EpCAM), fibronectin, developmental endothelial locus-1 (EDIL3), and Glypican-1 (GPC1) in exosomes were found to be significantly higher in BC patients compared to healthy individuals.^219,220^ Other proteins, such as HER2, CD47, Del-1, miR-1246, and miR-21, were also found to be significantly elevated in BC patients compared to healthy controls.^221^ Numerous studies have demonstrated that EVs may be used for diagnosing HCC. The complete analysis and screening of urinary EVs revealed that the glycoproteins LG3BP, PIGR, and KNG1 were highly elevated in these EVs obtained from the urine of HCC patients, but ASPP2 was dramatically reduced in these EVs.^222^ SMAD3, one of the molecules involved in HCC metastasis, also offers diagnostic potential for HCC, discriminating HCC from healthy to benign hepatoma^223^.

Similar investigations have been conducted in HNSCC, where altered expression of miRNAs, such as miR-186, miR-3651, and miR-494, has been observed in whole blood samples from HNSCC patients compared to healthy individuals. In HNSCC, altered expression of miRNAs such as miR-186, miR-3651, and miR-494 has been reported in whole blood samples from HNSCC patients compared to healthy persons. Salivary EVs from OSCC patients have also shown significantly higher levels of specific miRNAs, including miR-21, miR-184, miR-412-3p, miR-512-3p, miR-27a-3p, miR-302b-3p, miR-517b-3p, and miR-494-3p, compared to healthy controls.^209,212–214^ Furthermore, high expression of miR-486-5p, miR-486-3p, and miR-10b-5p has been observed in HNSCC cell lines.^224^ Increased expression of EGFR has been observed in LC cells, and higher levels of EGFR expression have been detected in EVs from LC patients compared to healthy controls.^210^ Studies analyzing patients with NSCLC have shown that elevated levels of exosomal miR-378 are associated with lymph node metastasis and advanced TNM stage, indicating its potential as a non-invasive prognostic biomarker.^211^ Furthermore, high expression of exosomal miR-146a-5p has been linked to cisplatin response, while miR-425-3p and miR-96 have been associated with cisplatin resistance in LC.^209^

Similar to this, miRNA-seq analysis of LCEVS from NSCLC patients identified three miRNA candidates (miR-320b, miR-10b-5p, and miR-15b-5p) for lung squamous cell carcinoma and four miRNA candidates (miR-181-5p, miR-30a-3p, miR-30e-3p, and miR-361-5p) for LUA as prospective biomarkers for early detection. The miRNAs let-7, miR-221, miR-137, miR-372, and miR-182 expressed in LC were shown to be potential biomarkers for predicting the survival rates of LC patients.^225^ Similarly, dysregulation of EV proteins, miRNAs, and lncRNAs has been observed in the serum of GC patients compared to healthy individuals, suggesting their potential as diagnostic markers for GC. Long noncoding RNAs, such as lncUEGC1,^226^ lncUEGC2, LINC00152, and lncHOTTIP,^227^ have shown significantly increased expression in exosomes derived from early-stage GC patients and could serve as biomarkers for predicting recurrence and progression at each tumor stage.^138^ MiR-21, miR-155, and miR-222 were identified as upregulated biomarkers in BC patients, showing promise for early and late-stage diagnosis.^228^ Established BC-associated protein markers such as CA 15-3, CA 125, and carcinoembryonic antigen (CEA) have been utilized for monitoring disease recurrence and metastasis.^229^ Overexpression of proteins such as human epidermal growth factor receptor 2 (HER2), EGFR, prostate-specific membrane antigen (PSMA), and EpCAM has been observed in various cancer types, including breast cancer, making them potential therapeutic targets and biomarkers.^230,231^ Studies involving extracellular vesicle-long RNA (exLR) sequencing of BC patients have revealed that exMSMO1 is significantly upregulated and may serve as a non-invasive biomarker for predicting treatment response.^232^ Liquid biopsies offer a non-invasive approach to monitoring advanced cancer and detecting tumor components in body fluids. Liquid biopsies can analyze various tumor biomarkers, including EVs, circulating tumor cells, circulating tumor DNA (ctDNA), circulating tumor RNA (ctRNA), circulating tumor proteins, and tumor-educated platelets.^233^ EVs derived from liquid biopsies provide valuable information about early diagnosis, cancer development, and progression in real-time for multiple cancer types [266]. In BC, several EV proteins have been extensively studied in many patient samples and show promising potential for early diagnosis. Proteins such as EDIL3, FAK, fibronectin, caveolin-1, Cyr61, ephrin type-A receptor 2, DnaJ homolog subfamily A member 1, polyadenylate-binding protein 1, and neuropilin-1 have been found to be expressed at higher levels in serum EVs of triple-negative BC (TNBC) patients compared to other BC subtypes, suggesting their relevance as biomarkers distinguishing between different BC subtypes.^233,234^ miRNAs in EVs have shown special potential as biomarkers for identifying HCC. EVs extracted from HCC patients’ serum showed that hsa-miR-483-5p was the only miRNA that was differently expressed in EVs from both HCC tissue and HCC patients’ plasma. MiR-483-5p was abundantly expressed in EVs of HCC and, through binding to CDK15 and suppressing CDK15 expression, increased HCC cell proliferation. Likewise, it has been demonstrated that miR-638 in EVs is a diagnostic for HCC diagnosis and an independent predictive factor for HCC patients.^222^

### Immune evasion and establishment of pre-metastatic niche by extracellular vesicles

**Fig. 4:**
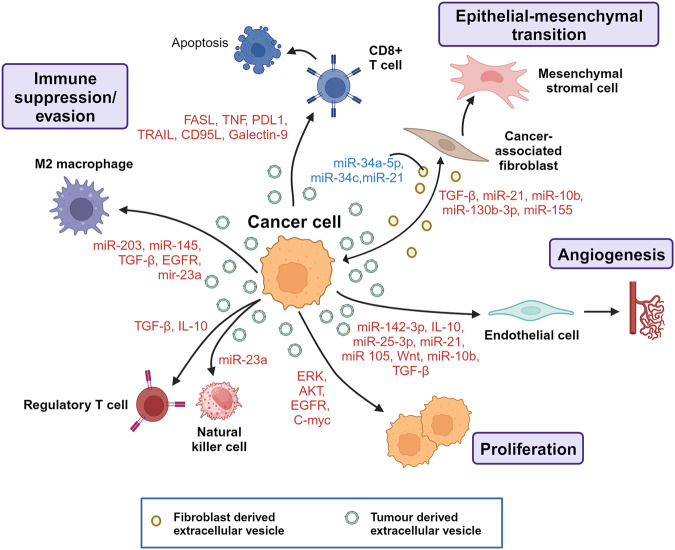
The multifunctional landscape of tumor-derived extracellular vesicles in tumorigenesis. This figure provides a detailed insight into the multifunctional activities of EVs emanating from cancer cells and their role in advancing tumorigenesis. Tumor-derived EVs encapsulate diverse bioactive molecules, including miRNAs, specific cytokines, and oncogenes. These molecular constituents determine the functional role of the EVs. EVs transport growth-promoting miRNAs and oncogenes to neighboring cancer cells, fueling their uncontrolled division and expansion.The EVs instigate a transformative process in epithelial cells by delivering specific miRNAs and proteins, endowing them with mesenchymal traits that enhance mobility and invasiveness.EVs convey pro-angiogenic factors to endothelial cells, stimulating the sprouting of new blood vessels, which nourish and support the expanding tumor mass. By presenting specific immunosuppressive cytokines and miRNAs to immune cells, such as macrophages, the EVs create an environment conducive to tumor evasion from immune surveillance. The EVs actively engage with various stromal cells, notably cancer-associated fibroblasts (CAFs) and macrophages. This cellular crosstalk, mediated by EVs, reshapes the tumor milieu, promoting a supportive scaffold and immune-tolerant backdrop for cancer progression

TDEVs have been demonstrated to influence non-cancerous and cancer cells, creating a TME conducive to tumor growth and metastasis. TDEVs impact cancer cells by promoting angiogenesis, which is crucial for tumor formation and metastatic spread, and by increasing vascular permeability, facilitating the dissemination of cancer cells.^235,236^ Additionally, TDEVs affect fibroblasts within the TME, driving their differentiation into pro-angiogenic and tumorigenic CAFs through various mechanisms.^237^ The functional oncoproteins carried by TDEVs induce phenotypic changes in recipient cells, activating and modifying downstream signaling pathways. These alterations create a supportive environment within the TME that fosters cancer growth, development, and metastasis. TDEVs also play a role in forming PMN, which support the growth of incoming cancer cells in distant organs.^238^ The lungs are the most frequently affected organs by metastatic tumors, followed by the liver, bone, brain, and lymph nodes.^239^ The cargo carried by tumor cell-derived EVs (TCEVs) may significantly impact PMN development more than the number of vesicles. Recent studies found that TSPAN proteins influence the TME through the increased proportion of stromal and immune cells in the TME.^240,241^ The cargo carried by tumor cell-derived EVs (TCEVs) may significantly impact PMN development beyond the number of vesicles. Upon reaching distinct organs, TDEVs deliver bioactive molecules, including miRNAs and proteins internalized by recipient cells.^242^ This internalization rewires the cells, preparing them for future metastatic growth and colonization (Fig. 4).^242^ The lungs are the most frequently affected organs by metastatic tumors, followed by the liver, bone, brain, and lymph nodes.^239^ Recent studies found that TSPAN proteins influence the TME through the increased proportion of stromal and immune cells in the TME.^240,241^ Metastatic tumors contribute to increased vascular permeability, an early mechanism in PMN establishment, often through endothelial dysfunction^243^ and destabilizing vascular adhesion between endothelial cells by destroying adhesion molecules.^244^ TDEV-derived miR-25-3p, for example, promotes vascular permeability and angiogenesis by inactivating KLF, a negative regulator of angiogenesis that reduces the promoter activity of VEGFR2 and maintains endothelial barrier integrity, such as destabilizing vascular adhesion between endothelial cells by destroying adhesion molecules.^243,245^ Moreover, miR-105 and miR-25-3p carried by TDEVs from breast or colorectal cancer cells can reduce the expression of tight junction proteins, leading to endothelial permeability, PMN formation, and subsequent metastasis in organs such as the liver, lungs, and brain.^246^

Tetraspanins, a group of plasma membrane proteins highly expressed in EVs, have been found to influence TME by altering the proportion of stromal and immune cells present.^240,241^ CD151, a tetraspanin, promotes tumor invasion in BC and prostate cancers and enhances trans-endothelial migration by inhibiting ERBB2 and overexpressing FAK, ERK, EGFR, and PKCα molecules. High CD151 expression is associated with a poor prognosis.^247–249^ Additionally, EVs carrying Sema3A on their surface contribute to elevated brain endothelial permeability in vitro and increased vascular permeability in vivo.^250^ TDEVs released by prostate cancer and mesothelioma cell lines express transforming growth factor β (TGF-β), which induces the differentiation of fibroblasts into myofibroblasts by activating the TGF-β/SMAD3 pathway.^251^

TDEVs carry soluble protein factors that influence the local ECM and stromal cells, contributing to the development of premetastatic niche conditions in target organs.^106^ Abnormal remodeling of the ECM, caused by tumor invasion, alters the characteristics of cancer cells by increasing matrix stiffness and providing substrates for their migration.^252,253^ ECM factors such as fibronectin, tenascin, periostin, and versican are involved in premetastatic niche formation. TDEVs stimulate recipient cells to remodel the ECM by triggering the release and rearrangement of these factors.^254^ MMPs, surface molecules found on TDEVs, play a role in ECM turnover and breakdown, facilitating ECM remodeling. TDEVs can modify the ECM to promote tumor growth by abnormal deposition or loss of ECM components.^255,256^ TDEVs induce the expression of MMPs in recipient cells at distant sites, favoring the growth of metastatic cancer cells. For example, TDEVs carrying HSP70 from metastatic tumor cells can induce the expression of MMP-2, which activates plasmin and further enhances metastasis.^257^ MMPs have also been shown to induce EMT by suppressing E-cadherin, leading to invasion and metastasis.^258^

The TME consists of various factors, and cancer cells interact with their microenvironment dynamically, including through cell-to-cell interactions, TDEVs, and cell-free interactions involving immune cells, stromal cells, and the ECM. TDEVs enable bidirectional communication between cancer cells and non-cancerous components within the TME. TDEVs primarily target CAFs, driving their differentiation into pro-angiogenic and tumorigenic CAFs through various mechanisms by delivering miRNAs and proteins.^259^ For example, TDEVs carrying miR-130b-3p have been found to activate fibroblasts, facilitating BC cell invasion and migration.^259^ MiR-130b-3p downregulates the expression of the SPIN90 protein, which is critical for maintaining stromal fibroblast characteristics^260^ and decreasing *α*-tubulin acetylation,^261^ which is necessary for myofibroblast differentiation.^261^ Additionally, miR-130b-3p inhibits tumor suppressive protein PTEN and activates mTORC1 in recipient cells through exosomal delivery, promoting tumor progression.^262,263^ TDEVs carrying miR-130b-3p can also accelerate LC development in vivo by targeting FOXO and activating mTORC1.^264^ Dysregulated expression of α-SMA,^265^ FAP,^266^ and CTGF^267^ is observed in myofibroblastic CAFs. CAFs, including those activated by TDEVs, promote cancer progression by secreting matrix-crosslinking enzymes, growth factors, and ECM components.^268,269^ Activated CAFs release MMPs, growth factors, chemokines, and ECM, which communicate with distinct sites and contribute to forming premetastatic niches.^270,271^ Modulation of the ECM can influence cancer progression by inducing cellular transitions, such as reducing the adherence characteristics of cancer cells and enhancing their motility and invasion.^272^ CAF-derived miRNAs, such as miRNA-34a-5p and miRNA-34c, induce EMT in OSCC by activating the AKT/GSK-3β/β-catenin signaling pathway, promoting cancer metastasis.^273–275^ Altered CAFs, in turn, modify the local environment to enhance tumor cell colonization and increase tumor metastasis through ECM production.

Furthermore, EVs also play a regulatory role in the immune system within tumors, suppressing immune cells and preventing the immune system from effectively detecting tumors. In HNSCC, EVs derived from IL-6-dependent inflammatory stimulation contain IL-10, which enhances angiogenesis, supporting tumor growth.^276–278^ In OSCC, miRNA-382-5p and miRNA-196a carried by EVs induce cell migration, further contributing to tumor progression.^275,278,279^ HNSCC-derived exosomes, when taken up by macrophages in the TME, trigger the proliferation, motility, and invasion of tumor cells by regulating the NF-κB pathway.^278^ Additionally, exosomes from the placenta have been implicated in promoting a state of immune privilege. Exosomes produced from the serum of pregnant women can modulate T-cell signaling during pregnancy by suppressing CD3-ξ and JAK3, potentially allowing for maternal-fetal immune tolerance.^280^

Tumor cells employ various strategies to exploit an immunosuppressive tumor environment, allowing them to evade immune surveillance and promote metastasis. EVs have been found to possess immunosuppressive activities and can impact both normal cells and cancer cells (Fig. 4). For instance, TDEVs carrying PD-L1 can inhibit T cell activation in vitro, contributing to tumor growth and progression.^281^ In laboratory studies, incubation of PD-L1-rich EVs with human CD8^+^ T cells resulted in immune suppression and reduced expression of CD69, a marker of T cell activation.^58,282^ In breast, lung, and thyroid cancers, exposure of activated CD8 + T cells to PD-L1-rich EVs suppressed the secretion of granzyme B, a protein involved in T cell activation and apoptosis.^59,283^ TDEVs enriched with molecules like CD95L, TRAIL, or galectin-9 can also promote T cell apoptosis.^284,285^ PD-1-expressing CD8^+^ T cells, when exposed to PD-L1-rich TME, undergo functional exhaustion and dysfunction. These cells release EVs that can impair the proliferation and function of normal CD8 + T cells (PD1-TIM3).^286,287^ Other immune cells are also affected by TDEVs. For instance, EVs derived from pancreatic ductal adenocarcinomas and containing macrophage migration inhibitory factor induce the secretion of TGF-β from Kupffer cells. TGF-β activation leads to HSC recruitment, contributing to tumor metastatic progression by promoting pre-metastatic niche formation and the recruitment of macrophages and granulocytes.^254^ Additionally, TDEV-derived RNA can activate Toll-like receptor 3 (TLR3) in lung epithelial cells, triggering the secretion of chemokines and promoting neutrophil recruitment, thereby creating a conducive environment for lung metastasis^288,289^ EV-derived RNA binding to endosomal TLR3 in DCs stimulates interferon production, which enhances the number of regulatory T (Treg) cells and supports tumor growth.^290^ Furthermore, EVs derived from ovarian cancer and enriched with FasL (Fas Ligand) on their plasma membrane induce apoptosis and suppress T-cell receptor/CD3-zeta expression in T lymphocytes. They also block the NKG2D-dependent cytotoxicity of NK cells, and CD8 + T cells.^291^ Immune checkpoint pathways have been identified as crucial targets for anticancer therapies, including TIM3, TRAIL, PD1/PD-L1, CTLA4/B7, and Fas/FasL.^292^ TDEVs enriched with immune checkpoint ligands can inhibit tumor killing by binding to T-cell cognate receptors. Recent studies have revealed that TDEVs can reprogram cancer-infiltrated dendritic cells (DCs) toward a tumor-promoting phenotype, thereby mediating tumor immune suppression.^293^ The maturation and function of DC cells depend on the composition of their contents, but TDEVs content, including HSP, TLR, HLA g,^294^ S100A8, S100A9, Annexin A1,^292^ PGE2,^295^ TGFβ1,^296^ or miRs,^297^ suppress DC maturation. Normal and tumor-derived EVs have been shown to carry and transfer proteins, lipids, and nucleic acids to neighboring cells. These EVs transport specific substances that can modify the metabolic profile of recipient cells in various ways, supporting tumor growth and potentially having systemic effects, such as cachexia.^224^ Proteomic analysis of EVs from different tissues has revealed the frequent expression of glycolytic enzymes in these vesicles. Furthermore, EV protein and RNA-Seq analysis have identified the enrichment of miR-155 and miR-210, which play a role in metabolic reprogramming, including glycolysis and oxidative phosphorylation.^298^

BCEVs carrying miR-105 have been found to induce metabolic reprogramming in CAFs. This activates a metabolic reprogramming signaling pathway in CAFs, enhancing glucose and glutamine metabolism to fuel adjacent cancer cells. CAFs utilize metabolic waste products such as lactic acid and ammonium under nutrient-poor conditions, converting them into energy-rich metabolites.^299^ Nasopharyngeal carcinoma-derived EVs packed with latent membrane protein 1 (LMP1) can transform fibroblasts into CAFs and increase aerobic glycolysis and autophagy. In vivo, LMP1-activated CAFs promote tumor growth and induce the expression of premetastatic niche factors in lung and liver tissues.^300^ In OSCC, EVs delivered by cancer cells can transform normal human gingival fibroblasts into CAFs. This transformation involves the degradation of caveolin-1 (CAV1) by activating the ERK1/2 pathway, leading to metabolic switching toward aerobic glycolysis in fibroblasts.^301^ Additionally, some cancers highly express glucose transporter 1 (GLUT1), and the transfer of secreted GLUT1 through EVs to recipient cells can contribute to metabolic changes in those cells. EVs derived from RB-associated cancers have also been shown to carry proteins involved in glycolysis, glucose catabolism, and amino acid synthesis. These cargos can reprogram cancer, neighboring stromal cell metabolism, and systemic energy metabolism.^302^

### Role of extracellular vesicles in chemoresistance

Resistance to cancer therapies remains a significant challenge despite efforts to target various dysregulated molecules. Resistance can arise due to multiple factors involving the host and tumor factors. EVs, abundant in the serum, have been shown to play a role in resistance to various anticancer therapies and have diagnostic significance. Notably, EVs derived from MSCs contribute to therapeutic resistance, including chemoresistance, targeted therapy, and immunotherapy resistance.^303^ Mechanisms underlying EV-mediated chemoresistance involve increased drug efflux, decreased drug toxicity, and enhanced DNA repair.^303^ One of the key contributors to chemoresistance is the overexpression of ATP-binding cassette proteins, particularly P-glycoprotein.^304^ EVs carrying transient receptor potential channel 5 (TrpC5) can induce the expression of the multidrug efflux transporter P-glycoprotein in recipient cells.^305^ MSC-derived EVs can interact with recipient cells, modifying their biological behavior and influencing the development of human disorders.^306^ Recent studies have demonstrated that MSCs promote chemoresistance in GC and leukemia cells.^307^ MSC-derived EVs activate the CaM-Ks/Raf/MEK/ERK signaling pathway, leading to multi-drug resistance in GC.^307^ EVs derived from normal and dysregulated MSCs and cancer cells carry proteins and RNAs that interfere with therapeutic approaches. Extensive research has been conducted on the involvement of EMT in tumor resistance. EMT-mediated signaling pathways, many of which are anti-apoptotic, and upregulation of drug efflux pumps contribute to drug resistance and share characteristics with CSCs.^308^ miR-155 has been shown to enhance EMT and CSC phenotypes, leading to drug resistance.^309^ Exosomes from cells overexpressing miR-155 exhibit significantly higher resistance to certain drugs than control cells.^309^ TDEVs carrying specific cargo can alter TGF-/SMAD signaling and upregulate EMT markers in ovarian cancer cells, leading to platinum resistance.^310^

Additionally, exosome-derived CAFs carrying the miR-21 gene can enhance chemo-resistance in neighboring cancer cells by suppressing apoptotic protease-activating factor 1 (APAF1), a direct target of miR-21.^311^ CAFs produce EVs in the TME, where exosomal miR-522 has been found to suppress ferroptosis in CAFs and other tumor cells. Moreover, miR-522 reduces the generation of reactive oxygen species (ROS), ultimately decreasing chemosensitivity.^312^ TDEVs and CAF-derived miR-130b-3p also play a potential role in suppressing ferroptosis. In melanoma cells, miR-130b-3p activates the Nrf2/HO-1 pathway, inhibiting elastin-induced ferroptosis.^313^ Aberrant protein sorting and exosome release in human ovarian cancer contribute to increased cisplatin (CDDP) export and drug resistance.^314^

Immunological treatments have significantly improved the clinical prognosis of many cancers and have become the standard approach. However, the emergence of resistance mechanisms and disease progression following an initial response remain significant challenges. Various strategies are being investigated to overcome immune therapeutic resistance induced by EVs in cancer.^315^ Although EVs have been recognized as tumor clinical indicators, they also directly spread resistance mechanisms among cancer cells.^315^ For example, exosomes can interact with activated NK-92 cells and reduce the effectiveness of adoptive NK cell therapy in patients with Acute myeloid leukemia (AML). Exosomes derived from AML patients caused a significant decrease in the expression of the NKG2D receptor on the surface of NK-92 cells, indicating that exosomes may impair the efficacy of activated NK-92 cells by delivering inhibitory ligands to NK-92 surface receptors.^316^ Inhibition of the NKG2D receptor reduces the cytotoxicity of NK-92 cells against AML blasts, as this receptor is responsible for triggering a cytotoxic and cytokine response against threats.^317^ In another study, exosomes derived from neuroblastoma markedly reduced the effectiveness of Dinutuximab in vivo, and Dinutuximab treatment altered the immune cell infiltration within the tumor, creating an immunosuppressive TME characterized by increased tumor-associated macrophages and decreased tumor-infiltrating NK cells.^318^

PD-L1, an immune checkpoint molecule, is expressed by various cell types, including tumor cells, monocytes, macrophages, NK cells, DCs, and activated T cells.^319^ Monoclonal antibodies targeting immune checkpoints, such as anti-CTLA-4, anti-PD-1, and anti-PD-L1, have been used in immune checkpoint blockade therapy. However, prostate cancer has been demonstrated to resist anti-PD-L1 therapy,^320^ and the underlying resistance mechanisms in patients are often unknown. PD-L1 expressed on exosomes derived from metastatic melanoma has been shown to inhibit CD8 + T cell activation and promote tumor growth, which can be reversed by anti-PD-1 antibody therapy.^321^ PD-L1 splicing variants released by patients’ cells competitively bind to PD-L1 antibodies, resulting in treatment resistance.^322^ Recent research has suggested that exosomes produced by tumors and regulatory T cells expressing CTLA-4 on their surface may interfere with the effectiveness of ipilimumab treatment, highlighting the potential of exosome-carried biomolecules as novel markers for detecting tumors and understanding therapeutic resistance^323^.

### Therapeutic applications of extracellular vesicles in cancer

**Fig. 5:**
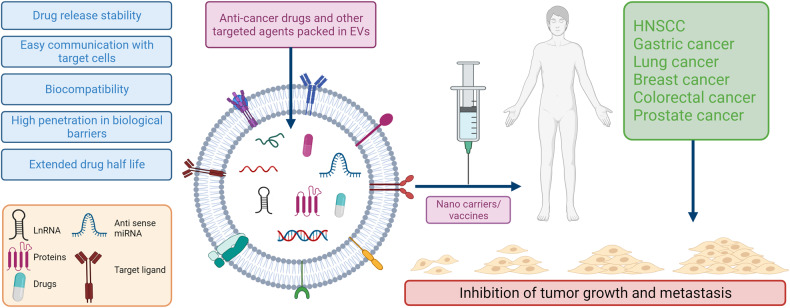
Extracellular vesicles for anti-tumor therapies. Extracellular vesicles (EVs) are gaining recognition for their potential therapeutic applications, particularly in oncology. Due to their inherent circulation stability and proficiency in mediating horizontal cargo transfer, EVs can be harnessed as carriers, loaded with various therapeutic agents ranging from chemotherapeutics to tumor-specific RNA interference molecules. Their natural ability to target specific cell populations makes them an ideal medium for precise drug delivery.EVs can be equipped with specific ligands to target malignant cells in diverse disease states, ensuring that therapeutic agents reach their intended sites with minimal off-target effects. By engineering EVs to display certain proteins, such as PD1 or tumor-specific antigen peptides, they can be transformed into tools for modulating the immune system. This strategy can amplify the body’s natural defense against cancers, potentially mitigating tumor growth or even initiating tumor regression. Researchers have devised innovative ways to repurpose EVs as vaccine platforms. By manipulating their content or surface properties, EVs can serve as promising candidates for next-generation vaccines, particularly for malignancies

Immunological treatments have become the gold standard for many cancers, significantly improving clinical prognosis. However, resistance mechanisms eventually develop, leading to disease progression in most patients. Various therapeutic strategies are being explored to overcome immune therapeutic resistance mediated by EVs.^324^ EVs have shown promise as tumor diagnostic indicators and are being investigated for their potential use in cancer therapy as vehicles for delivering therapeutic agents.^324^ One strategy is to target the biogenesis and secretion of EVs from cancer cells as a potential approach for cancer therapy. Rab proteins have been implicated in EV production by both normal and cancer cells.^325,326^ Inhibition of Rab27b reduces EV numbers and inhibits lung metastasis in BC cells.^327^ Additionally, inhibitors such as GW4869, which target nSMase2, have been shown to decrease EV secretion and metastatic rates.^328–330^ Modulating intracellular calcium levels with agents like dimethyl amiloride or blocking H + /Na+ and Na + /Ca2+ channels has also decreased EV release and delayed cancer cell growth in tumor-bearing mice.^331,332^ Targeting EV biogenesis and secretion holds potential clinical implications for metastatic cancer therapy. Another therapeutic approach is capturing and removing circulating tumor-derived EVs. Researchers have explored capturing circulating EVs derived from cancer cells using antibodies against human CD9 and CD63. Treatment with anti-CD9 or anti-CD63 antibodies enhances EV removal by macrophages, reducing tumor metastasis.^333^ Targeting surface proteins on EVs can also impede EV transport to distant sites, thereby preventing the formation of premetastatic niches.^28^ The roles of EVs in immunological reactions have been extensively investigated, and they have shown potential in drug development and therapeutic delivery systems. EVs possess properties such as biocompatibility and stability, and their expression of CD47 enables them to evade immune rejection, prolonging their circulation time compared to cell-based or free drug therapies.^17^ DC-derived EVs, enriched in membrane proteins involved in antigen presentation, have been used in phase I trials in melanoma, CRC, and NSCLC patients.^334–337^ These trials have demonstrated the safety of DC-derived EVs and their potential antitumor effects.^338,339^ Engineered EVs have gained attention as biomolecule carriers due to their efficient and safe delivery capabilities (Fig. 5). They have shown promising potential in cancer immunotherapy, enhancing therapeutic efficacy. For example, engineered EVs expressing monoclonal antibodies specific for T-cell CD3 and cancer cell-associated receptors have increased the anti-tumor activity of T cells.^340,341^ Engineered EVs carrying immune activators such as the STING agonist cyclic GMP-AMP have inhibited tumor growth and boosted anti-tumor immune responses^342^ EVs derived from CAR T cells have been investigated as an alternative to using the cells themselves in anticancer therapy, demonstrating efficacy with minimal side effects.^343,344^ Furthermore, EVs derived from human bone marrow MSCs (BMMSCs) have been enriched with specific miRNAs to inhibit glioma progression and promote BC dormancy.^345,346^ Synthetic miRNA mimics delivered through EVs have shown the potential to prevent migration and self-renewal of glioma cells and stem cells.^343^ EVs derived from human BMMSCs have been found to be enriched with specific miRNAs that exhibit therapeutic effects in inhibiting glioma progression and promoting cycling quiescence and early BC dormancy. In the case of glioma, low levels of miR-124 and miR-145 expression in glioma cells and germline stem cells (GSCs) make them suitable targets for miRNA replacement therapy in GBM. Administration of synthetic miRNA-124 and miRNA-145 mimics using BMMSC-derived EVs to glioma cells and GSCs has shown promising results in preventing migration and self-renewal, potentially hindering tumor progression.^347,348^ Similarly, BMMSC-derived EVs enriched with miRNA-199a and antagomiR-222/223 have inhibited glioma progression and stimulation of cycling quiescence and early BC dormancy, respectively.^345,346^ These findings suggest that delivering specific miRNAs using BMMSC-derived EVs could be a potential therapeutic strategy for targeting glioma and BC.

TEVs offer potential in the development of cancer vaccinations. These vaccines aim to utilize the “tumor-specific components” of TEVs to activate immune cells and restore immunological function, thereby creating effective tumor vaccines. One approach involves exploiting the natural response of tumor cells under heat stress to produce and release exosomes carrying heat shock protein 70 (Hsp70). In a study, myeloma cells were engineered to express Hsp70 on their cell membranes to mimic heat stress. The EVs derived from these modified myeloma cells contained a significant amount of Hsp70, which could activate immune cells such as CD11c+ dendritic cells (DCs) and CD4 + /CD8 + T cells, enhancing their chemotaxis capacity.^349,350^ Tumor-associated antigen (TAA)-enriched TEVs also hold great potential as antitumor vaccines in cancer therapy.^351^ EVs derived from renal cell carcinoma carrying TAAs were shown to elicit potent cytotoxic effects from CD8 + T cells against autologous tumor cells in vivo by mediating the Fas/FasL signaling pathway.^352^

Furthermore, DNA fragments derived from the phagocytosis of tumor cells by macrophages can activate STING signaling in neighboring DCs, promoting antitumor immunity.^353^ CD47 has also been explored in the context of TEVs by manipulating the immune checkpoint receptor. By blocking CD47 expression in tumor cells, TEVs preferentially accumulate and export tumor mitochondrial DNA, which activates STING signaling in nearby DCs, thereby enhancing antitumor immunity.^354,355^ Additionally, the administration of CD40L-enriched EVs (CD40L-EXO) into LC has effectively enhanced DC maturation and the activity of antitumor T cells. CD40 signaling is crucial in promoting DC maturation and the antitumor response.^356^ Moreover, EVs derived from dendritic cells can present tumor-associated antigens and induce T-cell activation, suppressing tumor growth. They can also trigger a humoral (B-cell)-mediated antitumor response.^357^ Overall, utilizing TEVs as components of cancer vaccines shows promise in activating immune responses against tumors and suppressing tumor growth. These approaches leverage the immunostimulatory properties of TEVs to enhance the antitumor immune response and hold potential for future therapeutic applications.

## Role of extracellular vesicles in diseases other than cancer

### Neurodegenerative diseases

**Fig. 6:**
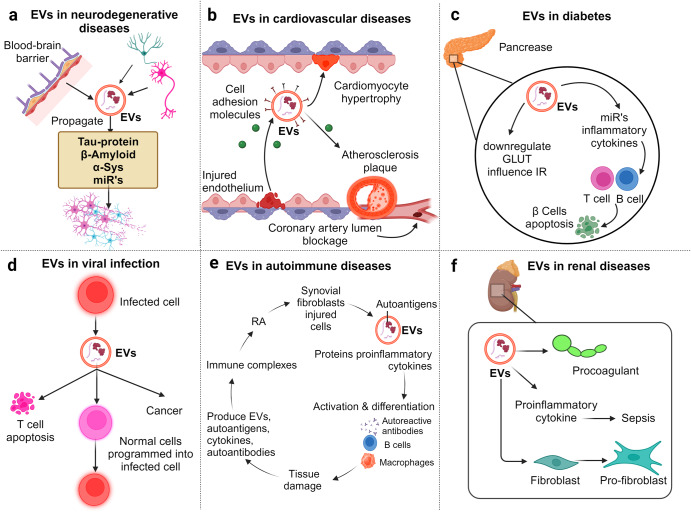
Pathological implications of extracellular vesicles in disease progression. **a** Neurological disorders. Cells such as microglia, astrocytes, and endothelial cells release EVs that transport neuropathological proteins like Aβ, Tau, and α-synuclein alongside specific miRNAs. These EVs can spread these harmful components across the brain, potentially accelerating neurodegenerative processes. Moreover, certain neurotoxic EVs can traverse the blood-brain barrier, disseminating their deleterious cargo to other neurons and amplifying neural dysfunction. **b** *Cardiovascular Complications*: EVs emanating from inflamed or damaged endothelial cells, as well as those from inflammatory cells, often display cell adhesion molecules and procoagulant markers. When the endothelium is compromised, these EVs adhere to the vascular wall, potentially instigating cardiomyocyte hypertrophy. Notably, patients suffering from acute coronary syndrome (ACS) demonstrate elevated concentrations of these circulating procoagulant endothelial microparticles. Over time, these contribute to atherosclerotic plaque formation, eventually risking blockage of coronary arteries. **c** *Diabetes*: In the diabetic milieu, EVs released within the pancreas carry detrimental miRNAs and pro-inflammatory cytokines. These EVs can instigate the apoptosis of insulin-producing β cells by activating specific immune cells, namely B and T cells. Furthermore, in type 2 diabetes (T2D), these EVs potentially reduce the efficacy of glucose transporters, exacerbating the disease. **d** *Viral Infections & Oncogenesis:* Cells infected by specific pathogens generate EVs that encapsulate viral components, facilitating their transfer to healthy cells. Some pathogens possess the capability to modify the content of EVs, leading to scenarios like T-cell apoptosis, potentially promoting oncogenesis. **e** *Autoimmune Diseases:* Physiological stress can spur the release of EVs enriched with autoantigens, inflammatory cytokines, and specific genetic elements (e.g., miRNA, DNA). These EVs can bind with antibodies and platelets to create immune complexes (ICs). These ICs then prime antigen-presenting cells, culminating in an intensified autoimmune reaction. This cascade can lead to heightened cellular destruction, as seen with the release of EVs from damaged synovial fibroblasts in RA. **f** *Renal Disease & Organ Interplay:* EVs circulating systemically facilitate communication between organs, and their dysregulated activities have been implicated in amplifying kidney damage and inflammation. These vesicles can potentially exacerbate renal ailments by fostering detrimental cellular interactions and inflammatory responses

Neurodegenerative diseases (NDD) are conditions that are characterized by the persistent loss of neurons, glial cells, and neural networks in the brain and spinal cord, as well as the selective malfunctioning of these cells. As a result, they can create different types of issues, such as ataxias, which are disorders of movement; dementias, which are disorders of mental functioning; and problems with breathing, speaking, and moving. Millions of individuals worldwide are affected by neurodegenerative disorders. The two most prevalent neurodegenerative illnesses are Alzheimer’s disease (AD) and Parkinson’s disease (PD).^358,359^ EVs serve various purposes in the nervous system, including intercellular communication, myelination maintenance, synaptic plasticity, antigen presentation, and trophic support for neurons.^359,360^ Along with their vital physiological functions in the central nervous system (CNS), EVs are also hypothesized to have a role in the etiology of neurodegenerative disorders (Fig. 6a). EVs have been shown to carry infectious prion particles that allow prion infectivity to propagate throughout cells in the nervous system. EVs have been linked to neurological disorders, which are distinguished by the gradual loss of neurons and usually associatively misfolded proteins. Misfolded proteins linked to various neurodegenerative illnesses, including β-amyloid and tau proteins in AD, α-synuclein in PD, and superoxide dismutase 1 in Amyotrophic lateral sclerosis, have been shown to use EVs to infect other cells.^361^ Neuron-derived EVs transfer toxic α-synuclein across neurons and non-neuronal cells, promoting the spread of PD. This buildup causes gradual neuron destruction, with oligomeric and polymeric forms being cytotoxic. α -Synuclein accumulation in glial cells can cause inflammation, which can then spread to neighboring neurons and glial cells.^362^ EVs in stroke patients are likely to reflect the proinflammatory character of the stroke, and as such, they can serve as a means for signaling CNS damage to the periphery. EVs from PD patient serum exhibit higher levels of inflammatory molecules, including IL-1 and TNF-α leading to α-synuclein and P62 aggregation, neuron degeneration, microglial activation, and apomorphine-induced abnormalities in mice.^363^ These aggregates may be hazardous through various processes, including endoplasmic reticulum stress, mitochondrial malfunction, disruption of axonal transport, synaptic toxicity, and loss of function of the aggregated proteins. Evidence shows that EVs have other roles, such as sequestering harmful oligomers or releasing them from protein aggregates.

Microglia are positively linked with tau pathology; however, how they play a role in tau propagation is unclear. Early in the disease, the hippocampus experiences an invasion of tau protein from the entorhinal cortex. It has been demonstrated that microglia-released EVs carrying β-amyloid and tau proteins contribute to developing and spreading AD by transporting these proteins to other cells.^364,365^ Interestingly, higher levels of EV tau were linked to poor memory performance but not to mood or behavior problems. EVs can cause constitutive tau proteins to change into their harmful versions when proteins like tau are released.^366^ Earlier, AD development was thought to be caused by interaction between brain cells.^367^ Numerous studies have found that EVs potentially transport harmful biomaterials inside the brain in recent years.^368^ For instance, endothelial cells, which are crucial parts of the blood-brain barrier (BBB), have been found to produce EVs that include β-amyloid (Fig. 6a) 343. The EVs with toxic Β-amyloid cross the BBB and cause mitochondrial malfunction and oxidative stress, which lead to a decrease in the generation of neurons from neuron progenitor cells. Rising Aβ protein levels disrupt the BBB in a cycle that results in an abnormal influx and outflow of β-amyloid containing EVs that promote neurodegeneration. A study found that in AD patients, brain-derived EVs increase the levels of hallmark proteins β-amyloid and tau and glial-specific molecules such as ANXA5, VGF, GPM6A, and ACTZ.^369^ In body fluid samples from AD patients, the combination of β-amyloid proteins, tau, and proteins unique to brain cells, such as ANAX5, VGF, GPM6A, or ACTZ, may be an applicant for a biomarker.^365^ Several neurological conditions are protein misfolding disorders, with evidence of harmful proteins spreading within the CNS. In inflammatory situations, microglia can also exchange signals with one another, which causes lipids to be released from microglia intracellularly and extracellularly via EVs to start the conversion of Β-amyloid from harmless insoluble forms to harmful soluble ones.^370^ Microglia-derived EVs showed cytotoxicity towards microglia and cortical neurons via transporting neurotoxic β-amyloid.^370^ EVs also serve as an essential source of biomarkers for such neurological problems, in addition to contributing to the pathophysiology of neurodegenerative disorders. An earlier study showed the potential of glioblastoma-derived EVs to serve as biomarkers for various neurological illnesses by harboring mRNAs and miRNAs. According to recent research, EV miRNAs like miR-44438 inhibit the degradation of α-synuclein in neurons, which causes their accumulation. In PD, miR-34a, which is released by astrocytes, encourages neurotoxicity and inflammation [335, 336]. Specific miRs (hsa-miR-23a-3p, hsa-miR-126-3p, hsa-let-7i-5p, and hsa-miR-151a-3p) have been shown in clinical investigations to dramatically decline in Alzheimer’s dementia (AD) when compared to controls.

### Cardiovascular diseases

Cardiovascular disease (CVD) is the leading cause of death, accounting for one-third of all fatalities worldwide in 2019. According to the WHO, 17.9 million deaths worldwide in 2019 were caused by CVDs, which accounted for 32% of all fatalities. There are four basic categories of CVD: aortic disease, peripheral arterial disease, coronary heart disease, and stroke.^371^ EVs have been identified as one of the important mediators of inflammation in a growing body of research on CVD (Fig. 6b). EVs also build up in human atherosclerotic plaques, significantly impacting biological processes such as cell proliferation, inflammation, blood clots, calcium deposits, and vasoactive reactions. Atherosclerosis (AT) is a type of CVD caused by continuous damage to the vascular endothelium and subsequent endothelial activation, apoptosis, and endothelial dysfunction, leading to atherosclerotic lesion formation.^372,373^ Since EVs can be produced by leukocytes, erythrocytes, smooth muscle cells, and endothelial cells, these vesicles are most likely the consequence of apoptosis or plaque cell activation. EVs may play an important role in the onset and development of atherosclerotic lesions due to their influence on blood clots, neoangiogenesis, cell survival, and endothelial integrity.^373^ EV triggers AT growth by initiating endothelial dysfunction, lesion formation, cell communication interference, inflammatory reactions, lipid deposition, calcification, unstable plaque progression, and injured plaque clotting and thrombosis.^374^ EVs cause myocardial damage and serve as myocardial ischemia (MI) biomarkers because of their increased release from activated or apoptotic cells.^375^ Cardiomyocytes release EVs in MI, rich in miRNAs like miR-143 and miR-222, in response to inflammatory conditions and low oxygen levels.^376^ MiR-146a-loaded endotheliocytes release EVs, which are messengers in the development of peripartum cardiomyopathy, which suppresses SUMO1 expression and induces cardiac dysfunction in maladaptive hypertrophy.^377^ Cardiac fibroblast-derived EVs enriched in miR-21-3p act as paracrine signaling mediators, promoting cardiomyocyte hypertrophy by targeting sorbin and SH3 domain-containing proteins.^378^ EVs can enhance communication and adhesion between blood cells and vessel wall-resident cells, potentially leading to athero-promoting and athero-protective effects. Endothelial cells (EC), erythrocytes, and vascular smooth muscle cells (VSMCs) are among the blood and vascular markers expressed by EVs found in human atherosclerotic plaques.^372,379^ Plaque EVs thereby encourage an unhealthy environment in the causing lesion favorable to plaque breakdown and proliferation.^380^ Plaque rupture triggers adverse events like thrombogenic material release, coagulation activation, platelet recruitment, and thrombus development in the wound area.^380^ The study found that αIIbβ3a integrin-bearing platelet vesicles (pEVs) are favored for interaction with platelets, fibrin, and the subendothelial matrix.^381–383^ Increased pEVs in high cardiovascular risk patients promote clotting on injured vessels. The researchers used an unbiased proteomic strategy to identify other platelet players that mediate blood-vascular cell interactions in thrombogenesis.^384–386^ They found dysregulation of proteins involved in cytoskeleton dynamics and cell adhesion processes, such as CUB domain-containing protein-1 (CDCP1) and kindlin-3 (FERMT3).^387^ CDCP1 activates β1-integrin and regulates adhesion in cancer settings, while FERMT3/Kindlin-3 regulates platelet integrin activation, adhesion, and aggregation.^388–390^ A transmembrane receptor called tissue factor (TF) is the main catalyst for blood coagulation. EVs positive for TF are spontaneously released from tumor cells into the blood. Tissue factor TF + EV may promote thrombus activation and migration of VSMC on eroding plaques.^391^ The interaction between P-selectin, leukocyte-derived EVs, and platelet adhesion molecule, which express PSGL1, enables the recruitment of active TF in the injured area and the dissemination of thrombus formation.^392^ EVs produced by activated endotheliocytes and inflammatory cells, particularly monocytes, which express cell adhesion and procoagulant molecules, indicate early vascular dysfunction.^393^ Procoagulant EVs are present close to high-risk susceptible plaques. They may help break apart the fibrous cap during atherosclerotic plaque erosion or rupture, exposing the ECM of the subendothelium to the bloodstream.^394^ The adhesion of platelet-derived EVs to the vascular wall is facilitated by injured endothelium, which in turn causes EC permeability and apoptosis, the latter of which is brought on by EV transfer of the activation of the TNF-α, caspase-3, and Rho-kinase enzymes.^374,395^ Recent studies found that CD144 EVs made exclusively from human endothelial cells correlates strongly with coronary endothelial dysfunction and that these levels are considerably increased in people with type 2 diabetes and atherosclerosis.^396^ Numerous diseases, including obesity, insulin resistance, and type 2 diabetes mellitus (T2DM), which are closely related to CVD, are associated with endothelial dysfunction, which manifests as a reduction in NO-mediated vasodilation.^397^ Evidence suggests that EVs from T2D patients reduce eNOS expression in cultured EC. It has also been demonstrated that EVs of endothelium origin reduce nitric oxide (NO) generation in vitro.^398,399^ Decreased expression and dysregulation of eNOS are connected with eNOS uncoupling, which is associated with CVD.^400^

Patients with peripheral artery disease (PAD) have shown elevated EV levels, which have been linked to atherosclerosis inside the peripheral vascular beds. PAD is expected to have significant consequences for society and the economy in the future since it raises the risk of cardiovascular events and death.^401^ Patients with acute coronary syndromes (ACS) have high levels of procoagulant endothelium microparticles in their blood, which have the potential to create and sustain intracoronary thrombi. A thrombus that forms on the contact of an atherosclerotic plaque that has burst or degraded causes ACS, a severe clinical symptom of coronary artery lumen blockage.^402^ As a result, in patients with coronary heart disease, the quantity of EVs produced from endothelial and platelet cells has been linked to circulating levels of IL-6 and C-reactive proteins.^403^ Additionally, medium-sized vesicles from individuals with ACS showed procoagulant activity in vitro; this activity ceased when cadherin suppressed phosphatidylserine.^404^ In summary, EVs are potent signaling molecules that can disrupt vascular homeostasis and cause vascular dysfunction, inflammation, plaque development, and thrombus formation.

### Diabetes

Diabetes is a chronic condition brought on by either insufficient insulin production by the pancreas or inefficient insulin use by the body. Insulin is a hormone that controls blood sugar. According to WHO reports, diabetes affected 422 million people in 2014, up from 108 million in 1980. Prevalence has been increasing more quickly compared to high-income countries in low- and middle-income nations. Diabetes is a significant contributor to renal disease, heart attacks, strokes, blindness, and lower limb amputation.^405,406^ Age-specific diabetes mortality rates increased by 3% between 2000 and 2019. An estimated 2 million people died from diabetes and renal disease in 2019. The pathophysiology of diabetes has been linked to EVs, which are released by cells in every part of the body. Type 1 diabetes mellitus (T1DM) and T2DM are the two classifications of diabetes diagnosis that are divided according to their mechanisms. The T cell-mediated autoimmune condition known as type 1 diabetes causes the loss of insulin-producing β cells in the pancreas (Fig. 6c). Type 1 diabetes is known to begin with lymphocyte invasion of the islets.^407^ EVs are regarded as a fundamental modulator of cellular communication and have been discovered to be increasingly essential in disease pathogenesis since materials exposed to cells in stress alter the survival of receiving cells. Understanding how EVs control cell survival and function may shed light on the etiology of diabetes and pave the way for creating more efficient screening and diagnostic technologies that might help catch the disorder early and stop it from worsening.^408^ In T1DM, impairment often develops due to the autoimmune destruction of islet β-cell mass and the subsequent targeting of β-cells inside pancreatic islets.^409^ As the condition worsens, β-cells start to die and become increasingly dysfunctional, which leads to insufficient insulin secretion and high blood glucose levels.^410^ Immunostimulatory EVs have been shown to induce the production of numerous cytokines, such as interleukin-6 (IL-6), IFN, tumor necrosis factor (TNF), and monocyte chemoattractant protein-1 (MCP-1), by activating T and B cells through a TLR4/MyD88-mediated pathway.^411^ EVs extracted from insulinomas can trigger the release of inflammatory cytokines in diabetic mice models via the MyD88-dependent pathway downstream of inflammatory signals from Toll-like receptors and interleukin-1. Activation of MyD88 can cause T-cell proliferation and activation, exacerbating the autoimmune response in T1DM patients.^412^ The miRNA content of EVs may play a significant role in the spread of T1DM pathogenesis. When EVs generated by cytokine-exposed cells were transferred to β-cells, they triggered apoptosis, which could be avoided by blocking Ago2 (a component of the RISC complex essential for miRNA activity).^413^ Chronic exposure to pro-inflammatory cytokines has been proven to disrupt cell communication and function in diabetes. A study revealed that pro-inflammatory cells derived EVs (cytokine-exposed EVs [cytoEVs]) induce cell dysfunction, promote a pro-inflammatory islet transcriptome, and boost CD8 + T cell and macrophage recruitment.^414^ A proteomic study of cytoEVs reveals an increase in the chemokine CXCL10 as membrane-bound to cytoEVs, allowing direct interaction with CXCR3 receptors on the cell surface. The binding of the CXCR3 receptor to CXCL10-cytoEV causes cell malfunction, inflammatory gene expression, leukocyte migration to islets, and antigen presentation.^414^ EVs generated by β cells also contain significant levels of intracellular autoantigens, such as the protein tyrosine phosphatase-like molecule IA-2 and the glutamic acid decarboxylase 65 kDa isoform (GAD65).^415^ Self-reactive T lymphocytes are activated, and β cell death occurs once the intracellular autoantigens are detected and taken up by the antigen-presenting cells (APCs). Higher EV expression of miR-21, miR34a, miR-146a, and miR-29 as a result of cytokine exposure early in the disease triggers cytokine-induced cell dysfunction.^416^ The expression of miR-375 and miR-148a-3p in EVs is linked to cell death. EV-carrying miR-142-3p/5p and miR-155 cause cytokine overexpression in pancreatic islet cells. A study showed that extracellular vesicular miRNAs from immune cells might encourage the malfunction and death of islet β cells. EVs produced from immune cells include the miRNAs hsa-miR-21-5p, hsa-miR-29b, hsa-miR-29c-3p, and hsa-miR-142-3p, which increase islet β cell death.^417^

A progressive insulin production deficiency, often known as insulin resistance (IR), and an insensitive bodily reaction to insulin cause type 2 diabetes. According to earlier studies, adipocytes, hepatocytes, and muscle cells could directly block the effects of insulin, which could result in systemic insulin resistance.^418,419^ Insulin resistance in T2DM results from insulin’s failure to control glucose metabolism in peripheral organs such as the liver, adipose tissue, and skeletal muscle. According to recent research, certain aberrant EVs from various cells may directly or indirectly produce insulin resistance by triggering inflammation, down-regulating glucose transporter type 4 (GLUT4), and influencing the insulin receptor (IR).^420^ It has been demonstrated that EVs from obese or diabetic mouse models can promote insulin resistance and glucose intolerance, whereas EVs from healthy and lean mice can correct these symptoms.^421,422^EVs have been demonstrated to include hormones and autoantigens that play a crucial role in insulin sensitivity and the development of the robust, deleterious autoimmune response in T1DM, such as insulin receptor substrate 1, GAD65, IA-2, and proinsulin. Adipose tissue-derived EVs can enter the brain in a membrane protein-dependent way, leading to synaptic disruption and accompanying cognitive impairment. Their cargo, miRNAs, are messengers between the brain and fat tissue. An alternative technique for pharmaceutical therapies to treat cognitive impairment linked to insulin resistance and T2D may involve targeting AT-EVs or their cargo miRNAs. In particular, synaptic injury and cognitive impairment are brought on by adipose tissue-derived EVs from animals fed a high-fat diet (HFD) or T2DM patients. Serum EV miR-9-3p levels were considerably more significant in people with obesity-related insulin resistance than in persons without diabetes, and they were much higher in diabetes patients with moderate cognitive impairment compared to those with normal cognition.^423^

### Viral diseases

It has been demonstrated that EVs are involved in several viral infections, with EVs secreted from infected cells that affect viral propagation. Most of the research revolved around Human immunodeficiency virus type 1(HIV) and Epstein-Barr virus (EBV), but pertinent findings for other viruses have also been gained. Additionally, virus-infected cells produce EVs carrying viral components, e.g., latent membrane protein 1 (LMP1) of the EBV, and viral miRNAs are frequently transferred to uninfected cells (Fig. 6d).^424,425^ When recipient cells ingest EVs generated from EBV-infected cells, their LMP-1 content can activate adhesion molecules like ICAM-1.^426^ In addition to LMP-1’s function in fostering infectivity, EVs from EBV-transformed cells stimulate the receiving cell’s ERK and Akt signaling pathways. It has been hypothesized that this stimulation of growth signals influences EVs’ capacity to cause tumors.^424^ EVs released from infected also carry host proteins. Galectin-9, a host protein released by EBV-infected cells, interacts with the Tim1 membrane receptor and causes T cells to undergo apoptosis (Fig. 6d).^427^ There is proof that vesicular galectin-9 can make EBV-specific CD4+ cells apoptotic, negatively affecting macrophage and T cell activation.^285^ The content of EVs can be altered by oncogenic viruses and viruses that can cause long-term persistent infections, which have been postulated to make infection easier and contribute to persistence and pathogenesis. The ability of these viruses to influence host cells without exposing viral proteins or virions to the immune system is due to the systemic circulation of viral proteins in EVs.^428^ For example, EBV-infected cells that express the viral glycoprotein gp350 mainly target B cells that express the viral entry receptor CD21 and can prevent naïve B cells from becoming infected by EBV by producing EVs that target these cells.^429^ EBV-infected cells discharge EVs containing EBV nucleic acids, precisely 44 miRNAs arranged in three clusters, namely the BHRF1-cluster, the BART-1 cluster, and the BART-2 cluster, which target the cell and viral miRNAs to elude the immune system.^430,431^ CXCL11, an IFN-inducible T-cell attractive chemokine, was revealed to be suppressed by BHRF1, while miR-BART15 was shown to control NLRP3 inflammasome activity and IL-1 production.^431,432^ The miR-BART15-3p was shown to be highly enriched in EVs from an EBV-positive GC cell line, inducing apoptosis in target cells, including immune system cells. As this cytokine is involved in antiviral activity, the downregulation of its expression reduces the antiviral response of the host cells.^433^

EVs have been found to be crucial to HIV replication and the challenges accompanying it. It has been shown that HIV modifies its pathogenicity by altering EV quantity and through EV-secretion mechanisms.^434^ EVs can increase cell susceptibility to infection by delivering viral receptors to cells lacking these receptors. For example, HIV protein Nef, released and transported via vesicular cells (EVs), can induce cell senescence or death in CD4 + T lymphocytes by decreasing CD4 incorporation into T cell EVs, preventing virions from binding to vesicular CD4 and increasing the circulating virus’s ability to infect cells. Similarly, HIV Nef, released with HIV, can induce cell senescence in CD4 + T lymphocytes by decreasing CD4 incorporation into T cell EVs, preventing virions from binding, and increasing virus circulation.^435^ Additionally, it has been demonstrated that EVs help viruses move from infected to uninfected cells, mostly by disguising viral antigens and PAMPs to prevent immune identification.^436^ It has been revealed that a significant amount of EVs generated by HIV-1 infected cells contain gp120 (Env), a viral protein that enables virus attachment and fusion to target cells as well as facilitates HIV infection in a variety of indirect ways. Viral infection of human lymphoid tissue ex vivo is reduced by the depletion of viral preparations of EVs, particularly those that carry gp120.^437^ The C-terminal amyloid precursor protein, or “C99,” prevents the gag from entering MVs during HIV-1 entrance. To counteract this, Gag promotes C99’s multi-site ubiquitination, which controls both the exocytic sorting of MVBs and the subsequent processing of C99 into dangerous amyloids. HIV Nef is released and transported by EVs, which cause bystander CD4 + T cells to undergo cellular senescence or death.^438^ Nef may reduce the quantity of CD4 incorporated into T cell EVs and degradation within the lysosomal compartment, preventing virions from attaching to vesicular CD4 and increasing the amount of virus in circulation that is available to infect the cell.^439,440^ Transfer of EVs with viral receptors to uninfected cells deficient in these receptors is another method that can render cells more vulnerable to infection, allowing these cells to become infected.

EVs produced from megakaryocytes and platelets can transfer the HIV co-receptor CXCR4 to a target cell lacking CXCR4.^441^ Similarly, EVs can transfer C-C chemokine receptor type 5 (CCR5) to CCR5 null cells from CCR5+ Chinese hamster ovary cells and peripheral blood mononuclear cells.^442^ Through the “unconventional secretion” of EVs, enteroviruses can leave cells without lysing them. Through the conveyance of en bloc virion, this alternate channel allows infection transmission from cell to cell.^99,443^ Many common human diseases, such as poliomyelitis (poliovirus; PV), hand, foot, and mouth disease (coxsackievirus; CV), and a recent respiratory infection outbreak of enterovirus D68 in the United States, are caused by enteroviruses.^444^ Dengue virus (DV) causes acute viral infections, typically by activating platelets and leukocytes, resulting in severe inflammatory responses such as cytokine storms and EVs secretion.^445,446^ Through the activation of CLEC2 and CLEC5A/TLR2 in platelets and leukocytes by DV and Influenza A *virus*. DV generates EVs that cause increased systemic vascular permeability and hemorrhagic shock, dramatically increasing disease severity.^447^ DV activates platelets via CLEC2 to release EVs, and DV-induced EVS further activates CLEC5A and TLR2 on neutrophils and macrophages, thereby inducing neutrophil extracellular trap (NET) formation and proinflammatory cytokine release.^448^

### Autoimmune diseases

Autoimmune disorders are caused by the interplay between genetic and environmental variables that trigger an immune response in the body to self-produced antigens. This causes self-damage to organs. Recent research has shown that EVs are potent immune stimulators, critical players in the etiology of autoimmune diseases, and both diagnostic and therapeutic biomarkers. In autoimmune disorders, immune cell tolerance mechanisms become troublesome, leading to the subsequent activation of autoreactive T and B cells.^449–451^ EVs transfer mediators, like proteins, cytokines, enzymes, and RNAs, can play a pro-inflammatory role on innate immune cells, affecting their activation, differentiation, and recruitment (Fig. 6e). EVs can facilitate the immune system’s presentation of self-antigens, mainly through the transfer of antigens from antigen-presenting cells (APCs) to autoantigen-specific T cells.^452–454^ The interaction of various inflammatory cytokines and chemokines can lead to an imbalance between regulatory and inflammatory cells, abnormal autoantigen clearance mechanisms, and antigen presentation, resulting in the development of autoimmune diseases (Fig. 6e).^455,456^ Evidence indicates that EVs with damage-associated molecular patterns (DAMPs) secreted from stressed or injured tissues significantly contribute to inflammation.^457,458^ It is known that CD8 + T cells and NK cells receive the antigens via EVs encapsulating DNA-binding proteins (DEK). This may result in improved immune system activation and more effective antigen presentation.^459^ EVs from T cells release MMPs from Fibroblast-like synoviocytes (FLS), degrading ECM proteoglycans and contributing to cartilage degradation in rheumatoid arthritis (RA) through an NF-κB-dependent mechanism.^460,461^ For the first time, it has been revealed that RA patients have much higher levels of CD3+ and CD8 + T-cell-generated synovial fluid (SF) EVs than osteoarthritis (OA) patients.^462^ These EVs may cause autoimmunity by acting as autoantigens and autoadjuvants, promoting the production of autoantibodies.^463,464^ EVs express self-antigens and peptide-MHC complexes implicated in autoimmune diseases, including HSPs, histones, and α-enolase, potentially acting as self-antigen sources and fibrinogen components and CD5 antigen-like precursors and triggering autoreactive T-cells in the setting of MHC, making them a significant player in the autoimmune response.^87,465,466^ EVs, including E3 ubiquitin-protein ligase TRIM21, Lupus La protein (SS-B), and Smith antigen (Sm), are found in salivary gland exosomes, suggesting their potential role as autoantigens in systemic lupus erythematosus (SLE) immune complex development.^467,468^

EVs can play a role in developing immune complexes because they carry self-antigens.^469,470^ Citrullinated proteins, like macrophage apoptosis inhibitory factor, are found in the EV membranes of FLS-produced EVs, promoting immune complex development.^459,466^ More immunoglobulin-carrying plasma EVs were detected in SLE patients than in healthy people, and, intriguingly, platelet-derived EVs have been found to have a significant role in autoimmune reactions in SLE.^467^ It has been revealed that serum-derived EVs isolated from SLE patients significantly increase cytokine production in healthy peripheral blood mononuclear cells, resulting in a proinflammatory response.^471^ Similar results have been observed when apoptotic endothelial microparticles are extracted from the plasma of SLE patients. IFN-γ and TNF-α levels in EVs from SLE patients are much greater than in healthy people, which raises the possibility that these levels could be employed as new diagnostic markers.^471^ It has also been found that blood-derived plasmacytoid dendritic cells and myeloid dendritic cells show increased expression of costimulatory surface molecules and proinflammatory cytokines, such as MHC-I, IL-6, TNF-α, and IFN-γ.^472^ In RA, irreversible tissue damage (cartilage erosion) is thought to be caused by synovial fibroblasts secreting a variety of matrix-degrading enzymes. Several of these enzymes have been found in association with EVs called MMPs,^255^ and tobacco smoke has been shown to stimulate the release of these proteolytic EVs from human macrophages.^473^ Hyaline cartilage may be effectively broken down by matrix-degrading glycosidases independently and in conjunction with MMPs.^474^ Hexosaminidase D glycosidase and glucuronidase were discovered in EVs released by synovial fibroblasts.^475,476^ Patients with RA may experience cartilage degradation because of EVs released by synovial fibroblasts. Studies have shown elevated platelet MV counts in circulation and inflamed joints due to EVs in plasma and SF.^462,477,478^ These EVs can cause coagulation and contain proinflammatory cytokines, contributing to fibrin deposition and joint inflammation.^477,479^ EVs isolated from the SF of RA patients have profound biological effects, including promoting B-cell-activating factor synthesis and increasing IL-8, CCL5, RANTES, CCL2, MCP1, IL-6, ICAM-1, and VEGF production by cultured synoviocytes.^480^ Synovial fibroblasts can activate various pathways, including NF-B, AP-1, JNK, and EVs, as well as T cells and monocytes.

Patients with SLE may have a higher risk of thrombosis and CVD due to the high concentration of EVs in their plasma. It’s interesting to note that SLE patients’ EVs differ from EVs from RA patients, systemic sclerosis (SSc) patients, and healthy people by having a particular protein signature.^481^ Compared to EVs from RA patients or healthy people, fewer EVs from patients with SLE have the makeup of EVs from healthy people, and more of them are marked for removal by immunoglobulins, complement, and other opsonizing molecules.^481^ This occurrence might be related to the SLE-specific impaired clearance of apoptotic bodies. The etiology of RA is believed to follow a similar pathway to that of SLE in terms of how EV contributes to inflammation. When compared to healthy people, patients with active SLE have higher levels of endothelial EVs, which can serve as indicators of endothelial dysfunction.^482^ Immunosuppressive medications were administered to these patients, which decreased the amount of endothelial EVs in the bloodstream and enhanced endothelial function. Because there are more circulating EVs in SSc patients, activated cell populations may interact through EVs, which may play a role in pathogenesis.^483^ High amounts of circulating EVs could significantly modify endothelial cell apoptosis, which has been thought to be a primary pathogenic event in SSc.^484,485^

### Renal diseases

EVs have a crucial function in the physiology and pathophysiology of the kidneys. Circulating EVs facilitate organ crosstalk and are implicated in the amplification of kidney injury and inflammation. In contrast, urinary EVs mediate crosstalk between glomerular and tubular cells and between various tubule segments (Fig. 6f). EVs molecular profiles, which indicate the type and pathological condition of the source cell, may be used for diagnostic and predictive reasons. The glomerular, tubular, prostate, and bladder cells are the most common sources of urinary EVs (99.96% proteins), primarily from urogenital tract cells. EVs were shown to have a role in the pathophysiology of the kidney by facilitating intercellular communication, transferring their content, activating signaling pathways in target cells, or simply serving as a channel for cellular waste disposal. EVs from upper tubule cells can be picked up by downstream cells by conveying active substances and altering the behavior of the receiving cells. A study found that murine kidney collecting duct cells can transfer functional aquaporin 2 (AQP2) through the release and uptake of EVs.^486^ Recent research indicates that EVs with high lipid raft composition and acidic pH microenvironmental conditions can influence EV uptake. EVs have been linked to the multi-organ dysfunction that characterizes sepsis and septic shock, including acute kidney injury (AKI). Patients with sepsis had higher numbers of EVs made by platelets and endothelial cells, which increased vascular reactivity in an experimental model. Disseminated intravascular coagulation increased endothelial cell-derived and leukocyte-derived MVs, which were positive for endoglin and PECAM-1. It was discovered that more circulating procoagulant EVs were linked to the most severe instances of meningococcal septicemia.^487^ MVs, particularly in patients with AKI and animal models, may contribute to organ failure during sepsis due to their procoagulant and proinflammatory properties. EVs that are released from red blood cells, platelets, and endothelial cells in patients with membranous nephropathy and minimal change nephrotic syndrome expose phosphatidylserine, which may increase the risk of thrombotic problems. The MV suspension isolated from plasma showed elevated tissue factor activity in patients with febrile urinary tract infections, which correlated with the disease’s severity. The plasma of patients with bacteremia had the greatest tissue factor activity, indicating that this factor may help to promote thrombosis during sepsis. In autosomal dominant polycystic kidney disease, polycystin 1 and 2 are reduced, and transmembrane protein-2 is increased, affecting kidney volume and interacting with the primary cilia of renal epithelial cells. Autosomal dominant polycystic kidney disease has been linked to reduced tumor suppressor miR-1 and miR-133a profiles in urine EVs.^488^ According to a study, EVs play a key role in controlling cyst formation in Autosomal dominant polycystic kidney disease (ADPKD) and provide evidence for the “cystic EVs theory,” which postulates that EVs from cystic renal epithelial cells may have an impact on the biology of nearby cells such as Pkd1 heterozygous renal epithelial cells, fibroblasts, and microphages. Upon being picked up by macrophages, EVs reprogram cells into a pro-inflammatory state, thereby accelerating the inflammatory cascade. EVs can contribute to kidney disease progression by causing inflammation in the tubulointerstitial. Animal models show that tubular epithelial cells release miR-23a-EVs in response to high HIF1 expression in an inflammatory environment.

Nephropathy is one of the major problems that almost one-third of individuals with diabetes experience. Although the pathogenic pathways are poorly understood, tubulointerstitial fibrosis is a characteristic of advanced diabetic kidney disease and is associated with a loss in renal function.^489^ All glomerular cells are affected, but podocytes are especially vulnerable to the effects of diabetic stress, including hyperglycemia and inflammation.^490,491^ Proteomic analysis of urine samples from nephrotic syndrome patients revealed the presence of proteins associated with proteinuria, like nephrin, TRPC6, INF2, and phospholipase A2 receptor1.^492^ Wilms tumor 1 (WT1) levels in urine EVs are significantly greater in patients with focal segmental glomerulonephritis and steroid-sensitive nephrotic syndrome, supporting podocyte contribution.^493^ Lysosome membrane protein 2 (LIMP2) was found in urinary EVs of membranous nephropathy patients, which was upregulated in patient glomeruli, suggesting that urinary EVs could serve as a potential biomarker for disease.^494^ Losing podocytes usually results in increased glomerular permeability and albuminuria development and is generally irreversible.^495–497^ Damaged human podocytes release EVs, causing a profibrotic phenotype in proximal tubule epithelial cells, promoting fibronectin and collagen IV expression, activation of p38, and SMAD3 activation.^498,499^ Puromycin-treated podocytes release EVs that induce tubule cell apoptosis through upregulated miRNA cargo transfer of miR-149 and miR-424.^500^ The reports summarized in this review highlight the growing importance of EVs in renal physiopathology, highlighting their complex functions in kidney pathophysiology and their impact on kidney health.

### Therapeutic potential of extracellular vesicles in other diseases

EVs play a crucial role in maintaining biophysiological homeostasis, cellular processes, and immune response. EVs are essential for cellular communication and drug delivery, promoting tissue regeneration, inflammatory regulation, and immune response. Also, EVs are crucial for several biological processes, including tissue remodeling, inflammatory resolution, repair, and regeneration. They prevent toxic substances and drug accumulation and can be used in chemotherapy and drug efficacy studies. They have been successfully applied in cancer therapy, inflammatory modulation, and immune response generation, making them a valuable tool for drug delivery and tissue regeneration.^22^ Stem cell-derived EVs (EVs) may offer cell-free treatments with therapeutic benefits, incorporating the therapeutic effects of their cells of origin. Stem cells can be pre-conditioned to produce and release EVs with varying therapeutic qualities. Due to their unique behavior, this makes them useful in clinical studies for treating various human disorders.^501^ Mesenchymal stem cell-derived EVs have been extensively researched in various fields due to their significant role as regeneration drivers. MSCs, due to their therapeutic effects, have gained attention for their secretory factors, suggesting that MSC-derived EVs could offer innovative therapeutic approaches. Modified EVs can be applied to loaded drugs, silencing RNA, miRNAs, and proteins for treatment (Table 3).

**Table 3:** Therapeutic applications of Extracellular vesicles

Molecule/Drugs	Source of EVs	Target model	Disease	Genes/Protein	Mechanism	References
EVs	Adipose tissue	Pancreatic islets C57BL/6J mice	Obese and Insulin-Resistance	GPCR, cAMP PKA	Enhances insulin secretion in T2D patients Mediators of obesity-associated Metabolic dysfunctions	^653,654^
Exosomes and EVs	MSCs HFL1	T2DM using an HFD and STZ Primary human skeletal muscle cells	T2DM	GLUT4, p-GSK3β, PCNA, Caspase3	Maintain glucose homeostasis Relieves β-cell destruction Reverses peripheral insulin resistance	^539,655^
EVs	Macrophages	miR-32^−/−^ mice, T2DM mice	T2DM	miR-32, Mef2d, cGMP-PKG	Autophagy inhibition of vascular smooth muscles	^656^
EVs carrying miRNAs	ADSCs	C57BL/KsJ db/m male mice	T2DM Nephropathy	TLR4, Bcl-2, caspase-3, Bax, IKKβ, IκBα, VEGFA	Protects Diabetic Nephropathy	^657^
Metformin	Urine	db/db mice on a high salt diet, mpkCCD cells	Diabetic Hypertension	cathepsin B, ENaC	Reducing high blood pressure	^658^
Dapagliflozin loaded exosomes	iPS-EC	Human iPSCs, iPS-ECs,	Diabetic Wound model	HIF-1α, VEGFA, C-X-C motif chemokine receptor 4	Facilitate angiogenesis Wound healing	^659^
EVs	Macrophage	M1 macrophage-induced HUVEC	Diabetic foot ulcer	miR-503, iNOS, Arg-1, IGF1R, ACO1	Regulates endothelial function affecting wound healing	^660^
EVs	Cardiomyocytes	ticagrelor-pretreated H9c2 cardiomyocytes HUVEC	Cardiovascular	Bnip3, Beclin, Bax, ENT1, miR-499, miR-133	Reduced hyperglycemia-induced ROS production Regulatory effect on diabetic cardiomyopathy	^661^
Empagliflozin	MSCs	H9c2 cells, Myocardial infraction animal model	Cardiovascular disease	miR-214-3p, Bcl-2, BAX	Inhibited myocardial apoptosis Reduced infarct size improved cardiac function	^662^
EVs	MSCs	Dendritic cells from CD14^+^ cells T1DM mice	Type 1 Diabetes	IL-10, IL-6, IFN-γ, FOXP3^+^ regulatory T cells, Th1, Th17	anti-inflammatory cell therapy for autoimmune disease prevention	^663,664^
EVs	2-E-EVs CoV-2	Vero E6 cells	COVID-19	nAb-1, nAb-2	Cell-to-cell transmission of SARS-CoV-2 Facilitates neutralizing antibody entry into cells	^665^
EVs	MSCs	COVID-19 patients THP1, Calu3, MVEC Rat model of LPS-induced ALI	COVID-19	ACE2, CD14, TLR4	Reduce morbidity and mortality Reduce lung inflammation	^666,667^
iNSC-EVs	Neural stem cells	NSCs, iNSCs	Alzheimer’s disease	GFAP^+^, Tuj^1+^ Iba^1+^, Aβ_1-42_	Therapeutic effects on cognitive function, neurodegeneration	^668^
EVs	Primary macrophages Neurons astrocytes	Neuronal cells, Parkin Q311X(A) mice	Neurodegenerative disorder	Tetraspanins, integrins	As nanocarriers for drug delivery to inflamed tissue	^669^
Vincristine (VCR)	Epidermoid carcinoma cells	KBv200 cells	Epidermoid carcinoma	ABCB1, Rab5, Rab8B	Increases sensitivity to chemotherapeutic drugs	^670^
Taxanes and Anthracyclines	Primary tumors BC cells	4T1 cells, C57BL/6, C57BL/6/Ccr2-KO mice,	Breast cancer	Anxa6, CCR2	Pro-metastatic Proinflammatory	^671^
Paclitaxel (PTX)	PTX- BC cells	HUVECs MCF-7	Breast cancer	circBACH1, miR-217, G3BP2 axis.	CircBACH1/ miR-217/G3BP2 axis a new regulatory for PTX-resistance and progression of breast cancer.	^672^
Cisplatin	Ovarian cancer cells	A2780, IGROV-1 cells	Ovarian cancer	CREB, ERK, JNK, p38α, p53	inhibits ovarian cancer	^673,674^
	Milk exosomes loaded with cisplatin	A2780, nu/nu mice	Ovarian cancer	ARF6, Rac1, CLTC, caveolin	Overcomes cisplatin-resistance in ovarian cancer	
Temozolomide	Glioblastoma cells	U87-MG cells, GBM cells	Glioblastoma	Heat shock protein, RAD51, MDM2	Leads to cell adhesion	^675^
EVs encapsulated miR-153-3p	LUAD cells	NCI-H1993, SW1271, BALB/c mice,	Lung adenocarcinoma	BANCR, miR-153-3p, PI3K/AKT	Increases cell invasion	^676^
EVs	PDAC cells	MIA PaCa-2, PANC-1,Rag2^−/−^Ilrg2^−/−,^ mice	Pancreatic ductal adenocarcinoma	Rab27a, LRP-4 receptor, YAP	Intratumor communication Targeted therapy for PDAC	^677^
EVs	Human Liver Stem Cells MSCs	Renal cancer cells SCID mice	Renal cell carcinoma	miR-Let7b, miR-200b, miR-200c and miR-223, EGFR, ZEB1, ZEB2, MMP1	antitumor effects	^678^

### Therapeutic potential in neurodegenerative disorders

EVs are exciting candidates as nanocarriers to treat brain disorders as they may be able to pass the BBB. EVs can potentially be exploited as a neurodegenerative disease treatment tool, addressing the brain via the BBB, boosting regeneration, delivering siRNAs and medicines, and restoring neurological functions. Yang et al. and colleagues found that bEND.3 cell-derived exosomes loaded with rhodamine 123 can pass the BBB and increase drug delivery to the brain. When loaded with anticancer drugs for treating neuronal glioblastoma (GBM), these EVs show a significant cytotoxic effect, demonstrating the potential of exosomes in overcoming barriers to drug entry.^502^ Therapeutic agents often struggle to reach adequate concentrations through systemic administration due to biological barriers, requiring intranasal delivery to bypass the BBB and directly reach the brain. For example, drugs are delivered to the brain directly through the intranasal route, avoiding the blood-brain barrier. One naturally occurring antioxidant and anti-inflammatory nutraceutical that has received a lot of research is curcumin. Intranasal delivery EVs containing curcumin, absorbed by microglia, show therapeutic benefits in various brain inflammation, autoimmune encephalitis, and tumor models, with significant tumor growth delay.^503^ Another promising approach to treating neurological conditions like stroke, traumatic brain injury, AD, and PD is stem cell-based therapy. MSC-derived EVs, administered intravenously in a post-stroke murine model, enhance neurological and spatial learning, reduce neurological deficits, promote angiogenesis and neurogenesis, and decrease inflammation.^504^ EVs produced from stem cells are thought to be intrinsic drug delivery systems and naturally occurring therapeutic agents for treating brain disorders. MSC-EVs have been shown to promote neurogenesis and angiogenesis in stroke patients, enhancing their functional recovery in vivo. Also, the therapeutic strategy for NDD involves reducing the pathological protein burden in the β-amyloid peptide (Aβ) metabolism alterations, which are the most rapid pathogenic event in AD development, occurring before clinical symptoms. The initial cleavage of the amyloid precursor protein to produce Aβ is catalyzed by BACE1 (β-secretase 1). As a result, blocking BACE1 activity may prevent one of AD’s early pathogenic processes. Mesenchymal stem-cell-derived EVs can deliver specific cargo to neurons, such as miR-29c-3p, which decreases BACE1 production while activating the Wnt/-catenin pathway.^505^ A study suggests EVs produced by transplanted stem cells can reduce β-amyloid and α-synuclein deposition, apoptosis, and oxidative stress while promoting angiogenesis and cell regeneration.^506^ According to a study, MSCs originating from adipose tissue release EVs containing neprilysin, which degrades β-amyloid peptides in AD.^507^ Additionally, the injectable hyaluronic acid hydrogel has been developed to deliver neural-stem-cell (NSC)-derived EVs into the stroke brain, enhancing EV retention and sustaining therapeutic effects, while induced NSCs from somatic-cells-derived EVs show comparable therapeutic effects.^508^ A study found that intraperitoneal injection of EVs from umbilical cord MSCs improved cognitive outcomes by decreasing neurological severity scores and improving reflex and sensation through an HDAC1-Dependent EGR2/Bcl2 Axis.^509^ It has been discovered that the EVs produced from human umbilical cord-derived MSCs (hUC-MSCs) has neuroprotective qualities against prenatal brain injury and lower the mortality of neuronal cells. In animal models of AD, intrathecal injection of MSC-derived EVs and iPSC-derived NSCs prevented microglial activation, enhanced synaptogenesis, and restored memory loss.^510^ EXs generated from hypoxia-preconditioned mesenchymal stromal cells can reverse cognitive impairment in the Alzheimer APP/PS1 animal model.^511^ The etiology of multiple sclerosis is significantly influenced by chronic inflammation. In Theiler’s murine encephalomyelitis virus model, intravenous treatment of MSC-EVs improved motor impairments and decreased brain atrophy. Furthermore, EVs demonstrated a significant reduction in plasma pro-inflammatory cytokine levels, allowing them to modify the activation status of microglia and reduce inflammatory infiltrates. In both in vivo and ex vivo, EVs extracted from IFNc-stimulated dendritic cells promote remyelination and reduce oxidative stress.^512^ The potential of immune-cell-derived EVs as therapeutic agents has been highlighted due to their capacity to modulate the immune system. Immunomodulatory effects and remyelination in EAE are produced by MSC-EVs coupled with LJM-3064 aptamer, a myelin-specific DNA aptamer exhibiting remyelination induction, on their surface.^513^

Stimulating astrocytes via inflammation and oxidative stress can induce them to release EVs, promoting recovery and potentially regenerating cells post-dementia injury. EVs can be used to transport therapeutic molecules for treating cognitive decline. Proteins like catalase can be packaged into EVs from macrophages and monocytes, reducing oxidative stress in PD.^514^ Research has also shown that engineered EXs, loaded with glial-cell-derived neurotrophic factors, have a strong neuroprotective effect.

### Therapeutic potential in cardiovascular diseases

Cell-derived nanocarriers EVs have low immunogenicity and toxicity compared to conventional nanocarriers. They are wrapped with unique biomolecules and endocytosed by target cells, delivering genetic information and protecting it from degradation, crossing biological barriers like the blood-brain barrier.^514^ The difficulty hinders the use of EVs in clinical practice in isolating enough EVs from culture systems and the heterogeneity of naturally occurring EVs. To overcome this, obtaining modified EVs has become a significant focus. These EVs are often derived from MSCs or induced pluripotent stem cells (iPS cells), both of which have positive properties mediated by their EVs. These EVs are proposed as potential starting points for cell-free therapy in nerve disorders. After MI, an engineered hydrogel patch was created that could slowly release and provide sustained delivery of EVs produced by iPSC-derived cardiomyocytes. This patch also promoted ejection fraction recovery, decreased arrhythmic burden, avoided cardiomyocyte apoptosis, decreased infarct size, and inhibited cell hypertrophy.^515^ A study on murine fibroblasts and iPS EVs found that both were enriched with angiogenesis-related miRNAs, hypoxic stress, cell cycle regulation, and aging processes. iPS-EVs contain unique miRNAs like let-7, miR-145, miR-17-92 cluster, and miR-302a-5p linked to cell proliferation, differentiation, apoptosis, self-renewal, and pluripotency.^516^ Recent research has shown that MSCs, obtained from various body tissues, have regenerative effects due to their complex secretion.^517,518^ Bone marrow MSC-EVs (BM-MSC) exhibit immunomodulatory properties, promoting anti-inflammatory macrophage polarization in infarcted mice through miRNA-182 delivery and TLR4 inhibition, significantly impacting cardiac repair. When EVs were produced from pro-inflammatory BM-MSCs by pre-incubating cells with low doses of lipopolysaccharide, as opposed to EVs from unaltered BM-MSCs, their ability to lower inflammation and trigger regenerative macrophage polarization was boosted.^519^ MSC-secreted miRNAs, including miR-21-5p, miR-146a, miR-30a, and let-7, have been found to regulate macrophage function.^520–522^ Under ischemic conditions, MSC-derived exosomes, loaded with miR-22, target Mecp2 in injured cardiomyocytes, preventing apoptosis and preserving ATP levels.^523^ In vivo studies showed improved cardiac function, increased systolic function, angiogenesis, and reduced apoptosis.^524^ The study investigates the positive effects of bone marrow MSC-derived EVs on primary neurons, revealing reduced iNOS (Inducible nitric oxide synthase) levels and improved cognitive behavior in APP/PS1 mice treated with EVs.^525^ EVs from pluripotent stem cells-derived MSCs promote angiogenesis in mice’s ischemic limbs. They enrich blood vessels with vascular endothelial growth factor (VEGF) protein and miRNA-210-3p, upregulating VEGFR1 and VEGFR2 expressions.^526–528^ Sun et al. found that hucMSCs are a source of MSCs, and their safety was established in healthy rabbits and infarcted rats. During the inflammatory phase, hucMSC-EVs promote fibroblast differentiation, reduce inflammation, and guard cardiomyocytes. The administration of bovine liver-derived catalase into mouse macrophage cell lines reduces reactive oxygen species (ROS) and microgliosis in a 6-OHDA lesions model in C57BL/6 female mice, promoting anti-inflammatory effects.^514^

### Therapeutic potential in diabetes

Due to their regenerative, anti-inflammatory, and immunomodulatory properties, EVs can potentially cure type 1 diabetes effectively. T1DM is an autoimmune condition in which auto-reactive T cells invade and destroy the pancreas’ insulin-producing β cells.^529,530^ EVs have been found to have immunomodulatory effects in T1DM, inhibiting reactive T cells and promoting regulatory T cells. They suppress auto-reactive T cells in animal models and inhibit APCs, Th1, and Th17 cell proliferation in vitro. In addition, EV treatment reduced inflammatory cytokines produced by reactive CD4 + T cells, including IFN-γ, IL-12, TNF-α, IL-6, and IL-17. In the early stages of type 1 diabetes, macrophages infiltrate pancreatic islets, causing inflammation and insulitis.^530^ In vitro, cord blood-derived stem cells can convert patients’ blood monocytes into M2 macrophages with anti-inflammatory properties. Numerous autoimmune diseases, including T1DM, are characterized by defective Treg-mediated immune control where FOXP3+ Treg number and function are diminished. A study showed that co-culture of human bone marrow stromal cells and peripheral blood mononuclear cells (PBMC) derived EXOs may inhibit the immunological response by enhancing Treg activation.^531,532^ Pancreatic cell regeneration is a potential T1DM therapeutic approach.^533^ MSC-derived EVs have been found to stimulate islet cell regeneration and insulin production by upregulating pancreatic and duodenal homeobox1 (pdx1), TGF-β, and smad1/2.^534,535^ TGF-β is essential for cell proliferation and differentiation.^536^ Pdx1 is involved in β-cell differentiation, survival, and functional maintenance. Similarly, it has been revealed that bone marrow transplantation in T1DM mice promotes β-cell regeneration by releasing EVs containing miR-106b-5p and miR-222-3p, which downregulate negative controllers of β-cell regeneration p21Cip1 and p27Kip1.^537^

T2DM is characterized by insulin resistance, impaired insulin secretion, and elevated glucose levels influenced by various organs and tissues. Although many studies have identified EVs as mediators of diabetes mellitus, EVs have also been shown to induce beneficial effects. Also, MSCs from various origins are desirable cells to separate EVs for therapeutic use in diabetic wounds. Stem cell-derived EVs can potentially enhance glucose tolerance and insulin sensitivity in diabetic individuals. The study is supported by using EVs in animal models to treat diabetes mellitus.^420^ EVs from adipose stem cells to treat obese mice found that the STAT3 protein carried by these EVs could induce macrophages to form anti-inflammatory M2 phenotypes through the transactivation of arginase, improving metabolic balance and insulin resistance.^538^ According to the study, miR-106b-5p and miR-222-3p found in EVs released by mouse bone marrow cells can stimulate the growth of pancreatic β cells by inhibiting the Cip/Kip pathway.^537^ EVs may be beneficial in treating type 2 diabetes, but further research is needed for clinical application. STZ-induced diabetic rats on a high-fat diet were given injections of EVs derived from hUC-MSCs, which partially reversed insulin resistance by improving cell destruction and indirectly accelerated glucose metabolism.^539^ The study found that EV treatment improved insulin receptor substrate 1 and protein kinase B phosphorylation in T2D rats, increased muscle GLUT4 expression, and maintained glucose homeostasis through liver glycogen storage.^538^ A study on diabetic foot ulcers in rats showed that EVs from Adipose tissue-derived stem cells (ADSCs) overexpressing NRF2 improved vascularization and wound healing.^540^ EVs effectively protect endothelial progenitor cells (EPC) during wound healing in high-glucose environments by reducing inflammatory cytokines and oxidative-stress-related proteins.^541^ The study found that EVs from UBC-EPCs promoted the angiogenesis of endothelial cells by activating ERK1/2 signaling.^541^

Similarly, it was found that EPC-derived EVs accelerated wound healing in diabetic rats by increasing endothelial cell proliferation and migration, thereby enhancing the levels of angiogenesis-related molecules like FGF-1, VEGF-A, VEGFR-2, and ANG-1.^542^ EVs derived from deferoxamine-stimulated human BMMSCs have accelerated diabetic wound healing by promoting re-epithelialization, activating angiogenesis, and developing collagen maturity.^543^ The study found that deferoxamine-EVs activate the PI3K/AKT signaling pathway through miR-126-mediated PTEN downregulation, stimulating angiogenesis in vitro.^543^ HSP90, STAT3, proangiogenic miRNAs (miR-126, miR-130a, and miR-132), and anti-inflammatory miRNAs (miR-124a and miR-125b) in human fibrocyte-derived EVs foster wound healing in diabetic mice.^544^

### Therapeutic potential viral diseases

EVs can transfer restriction factors produced by the host to adjacent cells, inducing antiviral reactions. Cells generate EVs carrying APOBEC3G (A3G), a cytidine deaminase that inhibits HIV-1 reverse transcription and prevents the virus from replicating.^440,545^ EVs with CD4 on the surface released by CD4 T cells can potentially limit HIV-1 replication and spread by functioning as a decoy for CD4 T cells and neutralizing HIV-1 virions, hence reducing HIV-1 replication and spreading.^546,547^ EV containing bacterial antigens derived from Mycobacterium bovis and tuberculosis-infected macrophages have been proven in studies to produce memory CD4+ and CD8 + T lymphocytes in the presence of dendritic cells, serving as a possible alternative vaccination approach for infectious illnesses. Studies in vitro have revealed that T cells can generate EVs expressing the HIV receptor CD4 that can bind to viral particles, thereby reducing the number of virions that would otherwise infect CD4 + T cells.^440,548–550^ As therapeutic carriers, EVs have numerous inherent advantages, including overcoming biological barriers such as the blood-brain barrier, a long circulation half-life, and immunostimulatory efficiency.^551,552^ Engineered EVs can be utilized for targeted drug delivery in antiviral treatments. The HIV-1 Nef adaptor protein is frequently expressed in EVs, playing crucial roles in viral proliferation and pathogenesis. Allowing viral antigens to fuse with mutant EVs anchoring HIV-1 Nef leads to the production of immunogenic EVs.^553^ The study suggests that a Nef-based engineering strategy could generate EVs for *hepatitis B vaccine (*HBV) immunization via cytotoxic T lymphocytes, potentially aiding in developing therapies against AIDS and HBV.^554^ HIV can counteract this by increasing HIV-Nef incorporation into EVs, inhibiting CD4 inclusion, and decreasing the efficiency of the host antiviral response. EVs produced using the Nef-based engineering technique have been shown to boost cytotoxic T cells’ ability to fight viral infection.

### Therapeutic potential in autoimmune diseases

EVs are being used as biomarkers, immunosuppressive or immunostimulatory agents, and as an alternative to mesenchymal stem cell transplantation. They can also be modified as nanocarriers for drug delivery systems, promoting tissue regeneration and repair, and as novel vaccines for tumor or infection treatment. Bioengineering techniques can also be used to modify EVs for specific nucleic acids, proteins, and therapeutic agents.^555^ BMMSC-EVs have the potential to be crucial mediators in cartilage repair, offering great promise as a new therapeutic for cartilage regeneration and OA. BMMSC-EVs, when co-cultured with OA chondrocytes, inhibit the adverse effects of inflammatory mediators on cartilage homeostasis. They upregulate COX2 and pro-inflammatory interleukins, inhibit collagenase activity, and promote cartilage regeneration in vitro. Furthermore, they stimulate the production of proteoglycans and type II collagen.^556^ Studies show MSCs-EVs can regulate chondrocyte hypertrophy markers MMP-13 and Runx2 in mouse chondrocytes while negatively affecting collagen type II alpha 1 chain, SOX9, and Aggrecan expression. Cell studies indicate that MSCs-EVs can significantly decrease IL-1 production, which is believed to reduce chondrocyte migration and death. EVs-circHIPK3 stimulated MSCs-EVs-mediated chondrocyte proliferation and migration while preventing chondrocyte death via the miR-124-3p/MYH9 axis.^557^ Data suggests that MSC-EVs show pro-regenerative, anti-apoptotic, anti-fibrotic, and anti-inflammatory properties in OA and RA, potentially improving cartilage regeneration in an immunocompetent rat model of osteochondral lesions.^558^ The study found that EV injections significantly accelerated neotissue filling, enhanced matrix synthesis of type II collagen and s-GAG, and led to the complete restoration of cartilage and subchondral bone.^559^ The second investigation demonstrated that EVs from synovium or MSCs from induced pluripotent stem cells effectively reduced the OA score in the CIOA model.^560^

### Therapeutic potential in renal diseases

Since most kidney disorders are incurable, they are hidden and complicated, making treatment extremely challenging.^561,562^ EVs, mainly stem cell-generated ones, have shown potential therapeutic properties in preclinical models of AKI, chronic kidney disease, and kidney transplant preconditioning.^563^ Mesenchymal stromal cells are widely used in regenerative medicine due to their proven immunomodulatory, pro-regenerative, and anti-inflammatory properties.^563^ BM-MSC EVs increased tubular cell proliferation and restricted apoptosis in models of toxic and ischemic AKI, which ameliorated kidney function loss and decreased plasma urea nitrogen and creatinine levels.^564–566^ Administering adipose mesenchyme stem cell-derived EVs improved kidney injury and reduced inflammation in cisplatin-induced and sepsis-induced AKI models.^567,568^ In cisplatin-induced AKI, miRNAs altered WNT-TGF, fibrosis, and EMT signaling pathways, whereas the protective mechanism in septic AKI involved elevated sirtuin 1 and decreased NF-κB levels.^567^

### Role of extracellular vesicles as nanocarriers

Both natural and synthetic EVs have shown promising potential therapeutic for a wide range of diseases. EV therapy is a good treatment strategy for various diseases due to its ability to transport cargo selectively and protect it from degradation. Significant progress has been made by using EVs as drug delivery vehicles and carriers of small molecules and gene therapies for cancer and potentially regenerative medicine in preclinical trials. Based on preclinical research, more than 50 clinical trials have been registered to assess EVs’ diagnostic potential as biomarkers for disease diagnosis.^569^ Many clinical trials are being planned and carried out (Table 4). Nanotechnology has significantly contributed to developing innovative carriers for targeted drug delivery, particularly lipid-based nanocarriers, which have led to the clinical translation of various formulations. However, these synthetic drug delivery systems have limitations due to inefficiency, cytotoxicity, and immunogenicity. Cell-derived EV-based carrier systems have also gained significant interest. Natural drug carrier systems, such as EVs, have been overgrown. EVs possess unique characteristics for drug delivery, making them promising for drug loading and targeted delivery. Understanding these EVs’ special features is crucial for maximizing their applications. Researchers focus on developing smart drug delivery systems with better targeting, safety, and pharmacokinetics than synthetic nanocarriers.^570^ Because of their ability to lower the hazardous effects of foreign drugs, EVs are preferred as drug carriers. They have a low immunological response, making them suitable for daily blood and plasma transfusions. Unlike virus-based cell therapies, EVs are non-replicative and non-mutagenic; therefore, regulatory difficulties are avoided.^571^ They possess unique qualities such as biocompatibility, low immune response, high stability, cell type targeting ability, and efficient drug transfer, making them attractive for therapeutic applications.^572^ These advantages are supported by low toxicity in vivo experiments. Blood-cell-derived EVs during blood transfusions have numerous safety features with no significant side effects, unlike platelet-derived EVs, which have been linked to transfusion-related acute lung injury.^573^ Efficient cargo loading of EVs as drug carriers requires a strategy for exogenous loading after EV isolation.^574^ Techniques like electroporation, simple incubation, sonication, extrusion, and freeze-thawing have been used, but they can result in aggregation and alter EVs’ characteristics.^575^ Endogenous loading, on the other hand, involves using cells’ sorting machinery to produce and load biomolecules, such as RNA or protein drugs, into vesicles.^576^

**Table 4:** Current extracellular vesicles based clinical trials for common diseases

S.No.	Disease	Type	EVs as a therapeutic target	Origin of EVs in clinical Use	Phase, patients	NCT	Refs.
1	HNSCC	HNSCC		Grapes	*P* = 1,*N* = 60	NCT01668849	^679^
		OSCC	Blood EVs	Metformin hydrochloride increases EVs	*P* = NA, *N* = 30	NCT02147418	
		Oral Mucositis Associated With Chemoradiation Treatment			*P* = 1, *N* = 9	NCT03109873	
2	Gastric Cancer		Circulating Exosomes		*N* = 80	NCT01779583	^680^
			Hsp70 carrying EVs		*P* = NA *N* = 71	NCT02662621	
		Ulcers (wound healing)	Plasma		*P* = 1, *N* = 5	NCT02565264	
3	Breast cancer		Detect EV induced Hypoxia		*N* = 21	NCT03262311	^681,682^
			Rosuvastatin reduces TFs		*P* = 2,*N* = 20	NCT01299038	
4	Lung cancer	Non-small-cell lung cancer lung metastases	Circulating EVs	DC	*P* = 2, *N* = 42	NCT01159288 NCT03108677	^683^
			RNA		*P* = NA, *N* = 90	NCT03108677	
	Liver caner	Advanced hepatocellular carcinoma and liver metastasis			*P* = 1, *N* = 9	NCT05375604	
5	Neurodegenerative disorders	Alzheimer’s disease Hypoxia-ischemia		MSC MSC	*P* = 1/2, *N* = 10	NCT04388982 NCT05490173	^683^
		Acute ischemic stroke		MSC	*N* = 300	NCT04202770	
				MSC	NA	NCT03384433	
6	Cardiovascular Diseases	Cerebrovascular disorders		MSC	*P* = 1,	NCT05043181	^683,684^
		Acute ischemic stroke		MSC	*P* = ½, *N* = 5	NCT03384433	
7	Diabetes	Insulin resistance T1DM T1DM T2D		Ginger MSCs Serum Serum	NA *P* = 2/3, *N* = 20 *N* = 20 *N* = 200	NCT03493984 NCT02138331 NCT04164966 NCT03264976	^684,685^
8	Renal Disease	Chronic kidney diseases		UC	*P* = 1, *N* = 20		^686^
9	Autoimmune diseases	Osteoarthritis Sepsis		MSC DC	*P* = 1 *N* = 50	NCT05060107 NCT02957279	^683,684^
10	Viral Infection	COVID-19 COVID-19		UC MSC	*P* = 2, *N* = 100 *P* = 2/3,*N* = 300	NCT04288102 NCT04392778	^687,688^

EVs have an inbuilt targeting ability because their membranes are loaded with ligands or receptors interacting with target cells. However, the vast majority of naturally produced therapeutic EVs are removed by macrophages, resulting in off-target effects.^577^ This problem is tackled by engineering EVs that are created by changing EV receptors to obtain targeted EVs. For example, EVs that have been modified with the GE11 peptide can target the EGFR, a highly expressed target in tumor cells, by binding specifically to other membrane proteins on their surface, such as PDGFR, acting as anchor points for targeting motifs.^578^ Following the encapsulation of paclitaxel, EVs containing aminoethylanisamide-polyethylene glycol exhibited enhanced anticancer effects, specifically targeting LC.^579^ EVs have intrinsic targeting properties for drug delivery, as their lipid composition and protein content can influence their tropism to specific organs. Regarded as EV surface indicators, tetraspanins have a role in cell adhesion and activation, forming a complex with integrin α4 or CD49d to target CD54-mediated endothelium and pancreatic cells.^580^ Different types of integrins can alter EVs’ pharmacokinetics, increasing their accumulation in the brain, lungs, or liver.^106^ EVs expressing Lamp2b have been successfully delivered to specific cells through siRNA-carrying dendritic cell-derived EVs fused to neuron-specific RVG peptides or muscle-specific peptides.^581^ A recent study used EVs from cardio-sphere-derived cells (CDC) for targeted delivery by fusing the N-terminus of Lamp2b to a cardiomyocyte-specific peptide (CMP).^582^ Selective gene silencing of PLK-1, a promoter of bladder cancer growth, was achieved by delivering PLK-1 siRNA using EVs as a transport vector to bladder cancer cells.^583^ EVs displaying single-chain variable segments effectively targeted tumor cells expressing a cognate antigen. The p88 expressing EVs carrying encapsulated plasmid DNA (pDNA) attach pancreatic β-cells preferentially and transmit (pDNA), indicating a quick internalization process for peptide-bearing EVs.^584^ Bioengineering techniques have been employed to create EVs with enhanced capabilities, and various anticancer drugs, including paclitaxel, curcumin, doxorubicin, celastrol, and elemene, have been encapsulated in EVs to improve drug bioavailability and inhibit tumor growth and metastasis.^572,585^ Chemotherapeutic agents such as doxorubicin (DOX), paclitaxel (PTX), methotrexate (MTX), tirapazamine (TPZ), cisplatin (Cis), imperialine, and nischarin can also be delivered through EVs.^586^ For instance, paclitaxel can be loaded into EVs derived from autologous prostate cancer cells, enhancing its cytotoxic effect on prostate cancer.^587^ In a study, superparamagnetic iron oxide nanoparticles (SPIONs) and curcumin were loaded into exosomes, and the exosome membrane was conjugated with a neuropilin-1-targeted peptide for glioma-targeted delivery, demonstrating potent anti-glioma effects.^588^ Tumor necrosis factor-related apoptosis-inducing ligand (TRAIL) engineered EVs loaded with triptolide were used for targeted delivery in malignant melanoma, resulting in the inhibition of proliferation, invasion, migration, and induction of apoptosis.^589^ Antibody-drug conjugates (ADCs), a new generation of anti-cancer medications, can be attached to specific antigen molecules expressed on the surface of TDEVs. This enables the targeted delivery of ADCs to tumor sites or even non-malignant cells within the TME, mediating anti-cancer effects.^590^ EVs carrying KrasG12D siRNAs or shRNAs were designed to target oncogenic KRAS in pancreatic cancer, suppressing oncogenic KRAS and increasing survival in mouse models.^591,592^ To enhance the delivery of anti-apoptotic Bcl-2 antisense oligonucleotide G3139 into tumor cells, cell-penetrating polypeptides were attached to the plasma membrane of EVs.^593^ In BC mouse models with strong EGFR expression, injection of let-7a-loaded GE11-positive exosomes significantly decreased tumor diameter.^594^ In summary, EVs can be engineered to deliver a variety of therapeutic cargoes, including anticancer drugs, siRNAs, shRNAs, peptides, and antibodies. Their potential in targeted drug delivery holds promise for improving treatment outcomes in various cancer types.

## Conclusion and future prospective

The identification and understanding of EVs and their role in human pathophysiology, particularly in cancer, have emerged as a promising area of research. EVs are produced by various cell types, including cancer cells, MSCs, infected cells, plants, microbiota, etc., and can be found in bodily fluids. They play a critical role in cell-to-cell communication, potentially even across different species, and are involved in various physiological and pathological processes such as angiogenesis, inflammation, tumor growth, and tissue degeneration. EVs carry diverse biological components resembling their parent cells and can influence recipient cells in normal and pathological conditions. However, due to the complex composition of EVs and their dual functions in biological processes and disease pathophysiology, there are still challenges in accurately classifying pathogenic and non-pathogenic EVs. Exploring and analyzing the genomic profiles of EVs holds promise for assessing and identifying different biomarkers for the early diagnosis of different diseases.

Additionally, EVs have been found to have both immune activation and immunosuppressive effects in TME. They are crucial in establishing premetastatic niche formation through immunosuppression, angiogenesis, stroma remodeling, and metabolic reprogramming in cancer. However, the field of EV biology, including its content, function, targeting, internalization mechanisms, and biomarkers, is still in its early stages of development, mainly due to technical limitations in detecting, isolating, and characterizing diverse populations of EVs, including small vesicles.

In recent years, there has been a significant increase in research on plant-derived EVs, which are gaining attention for their potential use as delivery vehicles for therapeutic compounds. Furthermore, studies have shown that EVs derived from the host gut microbiome can enter the bloodstream and spread to distant organs and tissues. The role of bacterial EVs in carcinogenesis is being explored, and further research is needed for cancer diagnostics and bioengineering strategies for cancer therapy.

EVs hold promise in the realm of therapeutics owing to their biocompatibility, minimal immunogenicity, and intrinsic stability. These properties make them superior to certain cell-based regenerative treatments, which might pose safety and viability concerns. While some EVs have advanced to clinical trials for disease management, the journey to large-scale production is fraught with hurdles such as mechanical damage to EVs and potential contamination.

One major limitation in the current EV landscape is the absence of standardized and reliable methods for mass-producing EVs from a consistent source. This is coupled with challenges in quality assurance, which is imperative when EVs serve as medicinal agents or vectors—ensuring the correct dosage and maintaining stability after extensive purification becomes pivotal. Storage environments that preserve the structural and functional integrity of EVs are crucial to harness their therapeutic potential.

Safety is paramount, and understanding any adverse ramifications of introducing EVs into the human body is a significant concern. Moreover, if EVs are to revolutionize drug delivery, we need to address several technological bottlenecks. These encompass enhancing the loading capacity of therapeutic vesicles, evading premature clearance from the bloodstream, precision targeting of specific cells, and ensuring the effective release and uptake of the therapeutic load within target cells.

To truly leverage the multifaceted benefits of EVs, especially their capability to ferry diverse cargos within a singular package, it’s imperative that the scientific community invests in both fundamental and applied research to navigate these challenges. As EVs naturally gravitate towards certain cells, engineering them to enhance this innate specificity will be pivotal. Tailored production of EVs for specific cellular targets will further propel their therapeutic prospects.

The burgeoning interest in EVs, stemming from their inherent therapeutic attributes and malleability for specific purposes, has ignited a wave of research into maximizing their therapeutic outcomes. To tap into their full potential, innovative strategies that address rapid clearance and ensure targeted distribution will be quintessential. EVs offer advantages as nanocarriers for various therapeutic applications compared to traditional synthetic carriers, opening new avenues for drug delivery. However, challenges related to high-purity isolation and large-scale production of EVs need to be addressed to fully harness their potential as therapies and vaccines. Further research is needed to overcome obstacles associated with engineering EVs for specific applications. Advancements in EVs engineering suggest a promising future for disease treatment, particularly when combined with traditional and engineering approaches that complement each other. These vesicles display benefits similar to those of their parent cells but with less toxicity, such as decreased immunogenicity and tumorigenicity. The toxicity of these vesicles may differ depending on the cell type and target disease; hence, preclinical investigations based on the specific vesicle system are required. In the future, we intend to develop a more generic and powerful evaluation method or model that can identify the general toxicity, immunogenicity, and tumorigenicity of diverse cell-derived EVs. Additionally, cytokine release syndrome (CRS) is a significant safety risk for using EVs in cancer immunotherapy. There is reason for hope that EV-based medicines will be quickly translated into clinical practice because clinical-grade EVs are created for anticancer therapies in huge quantities under standards. For improved immunotherapy, antigen loading effectiveness must be improved overall. Other challenges to be solved for getting EVs into clinical settings include the safety of manufactured EVs to minimize CRS, the elimination of microorganisms, and GMP standard requirements. A practical alternative to traditional medicines, cancer immunotherapy, has shown impressive clinical results in treating several malignancies. Regarding possible cancer treatments, immune cell-derived EVs are receiving a lot of interest. In laboratory and preclinical investigations, EVs produced from immune cells have effectively treated solid and nonsolid malignancies.

Finally, with on-going modification and improvement, as well as subsequent regulatory approval, EV compositions derived from various sources might be effectively used to treat one or more disorders. As a result, they may significantly contribute to therapeutic tools in the future. Future efforts should focus on developing methods to control EV release and disrupt cell-to-cell communication mediated by EVs. Extensive research is still required to fully understand the potential applications of EVs, unravel the mechanisms of EV secretion, and translate experimental findings into more effective clinical therapies.
